# Marek’s disease virus-encoded microRNA-M6-5p facilitates viral latent infection by targeting histone demethylase KDM2B

**DOI:** 10.1128/jvi.02007-24

**Published:** 2025-01-22

**Authors:** Linyi Zhou, Runan Zhu, Bo Jiang, Jing Cheng, Wenxiao Liu, Yongxiu Yao, Yongqing Li

**Affiliations:** 1 Institute of Animal Husbandry and Veterinary Medicine, Beijing Academy of Agriculture and Forestry Sciences 656308 , Beijing, China; 2 Beijing Key Laboratory for Prevention and Control of Infectious Diseases in Livestock and Poultry, Beijing, China; 3 Sino-UK Joint Laboratory for Prevention & Control of Infectious Diseases in Livestock and Poultry, Beijing, China; 4 College of Animal Science and Technology, Beijing University of Agriculture 74684 , Changping, Beijing, China; 5 The Pirbright Institute and UK-China Centre of Excellence for Research on Avian Diseases, Pirbright, United Kingdom; University of Virginia, Charlottesville, Virginia, USA

**Keywords:** MDV, latent infection, miRNAs, epigenetic regulation, KDM2B

## Abstract

Marek’s disease virus (MDV), a highly contagious and oncogenic avian alphaherpesvirus, establishes a latent infection primarily in CD4^+^ T cells. Latent infections are necessary for both persistent lifelong MDV infection and viral tumorigenesis. MicroRNAs (miRNAs) play critical roles as post-transcriptional regulators of viral infections. However, the role of miRNAs in regulating MDV latency remains unclear. In this study, we found that an MDV-encoded miRNA, miR-M6-5p, inhibited viral lytic replication *in vitro* by functional screening and that infection with an MDV mutant lacking miR-M6-5p resulted in impaired MDV latency, proliferation, and tumor formation *in vivo*. Importantly, we identified lysine-specific demethylase 2b (KDM2B), an important epigenetic factor, as a target of miR-M6-5p. Furthermore, KDM2B knockdown increased the level of the transcriptionally repressive histone mark H3K27me3 on the key lytic gene pp38 promoter, accompanied by suppression of pp38 expression and reduced latent-to-lytic switch in MDV-latently infected cells, while treatment of cells with H3K27me3 inhibitors (GSK126 and Tazemetostat) markedly promoted the expression of pp38 and MDV reactivation from latency. Thus, miR-M6-5p facilitates MDV latency by epigenetically suppressing pp38 expression by targeting KDM2B. These ﬁndings unravel the mechanism by which a virus-encoded miRNA plays a critical role in the regulation of latent MDV infection.

**IMPORTANCE:**

Similar to other herpesviruses, MDV can establish a lifelong latent infection in the host. During the latency, MDV integrates its genome into the host genome to maintain the viral genome, which is considered a prerequisite for tumor formation. Reactivation of the latent viral genome in response to intracellular and extracellular stimuli re-enters lytic replication, resulting in pathological recurrence and/or viral shedding. However, the regulatory mechanisms underlying MDV latency remain poorly understood. In the present study, we investigated the role of virus-encoded miRNAs in MDV latency. We found that miR-M6-5p facilitated MDV latency, proliferation, and tumor formation *in vivo*. Mechanistically, miR-M6-5p epigenetically suppressed the expression of the viral lytic gene pp38 by directly targeting the histone demethylase KDM2B. These ﬁndings will advance our understanding of the role of virus-encoded miRNA in the regulation of viral latency and will help guide the development of novel strategies for the effective control of MDV.

## INTRODUCTION

Marek’s disease (MD) is a highly contagious, immunosuppressive, and neoplastic avian disease that is widely prevalent and causes considerable economic losses in the global commercial poultry industry (1). MD virus (MDV), the etiological agent of MD, is an alphaherpesvirus that shares several oncogenic properties with human oncogenic herpesviruses, such as the Epstein-Barr virus (EBV) and Kaposi’s sarcoma-associated herpesvirus (KSHV), and is therefore considered a good model for studying herpesvirus-induced lymphoma (2). After MDV enters the host *via* the respiratory tract, the infection process consists of four phases (3). The first phase is early cytolytic infection within 3–7 days post-infection (dpi), which occurs mainly in B lymphocytes with massive viral particle replication (4). At approximately 7 dpi (second phase), MDV establishes a latent infection, mostly in CD4^+^ T cells, by integrating its genome into host telomeres. During latency, the viral cytolytic replication is restricted with limited viral gene expression (5, 6). In the third phase, MDV is reactivated from CD4^+^ T cells in response to intracellular or extracellular stimuli, which is accompanied by a second cytolytic infection and immunosuppression at approximately 18 dpi (7). Finally, there is a proliferative phase at approximately 28 dpi, characterized by the formation of visceral tumors derived from CD4^+^ T-cell lymphomas (8). In the MDV life cycle, latent infection is considered necessary for tumor transformation (9) and allows the viral genome to remain in the host for life and become a new source of infection through reactivation (10). Thus, latent infections play an important role in the pathogenesis and prevalence of MDV. Although several studies have revealed the events involved in the regulation of MDV latency, including histone modifications (11), DNA methylation (12), and viral integration (13), the regulatory mechanisms underlying the establishment and maintenance of MDV latency are currently poorly understood.

MicroRNAs (miRNAs) are 20–25 nucleotide single-stranded noncoding RNAs. The miRNA-mediated silencing complex (miRISC) formed by miRNAs and proteins, such as AGO2, recognizes and binds to the target mRNA through base complementary pairing and then achieves post-transcriptional regulation by degrading the mRNAs of the target gene or inhibiting translation (14, 15). For herpesviruses, virus-encoded miRNAs are among the few viral products that can be highly expressed in the latent infection phase (16–18). MDV-encoded miRNAs can be classified into three groups based on their location in the genome (19, 20). Cluster 1 miRNAs, also known as the Meq cluster, are located upstream of the meq gene and include the miRNA precursors miR-M2, miR-M3, miR-M4, miR-M5, miR-M9, and miR-M12. Meq cluster miRNAs, particularly miR-M4, are required for MDV-induced tumor formation (21–23). Cluster 2 miRNAs are located between cluster 1 and cluster 3 miRNAs, also known as the middle (mid) cluster, and consist of the miRNA precursors miR-M1, miR-M11, and miR-M31. Deletion of mid-cluster miRNAs increases mortality in chickens infected with MDV (24). Cluster 3 miRNAs are located upstream of the latency-associated transcript (LAT), also known as the LAT-cluster, and include the miRNA precursors miR-M6, miR-M7, miR-M8, miR-M10, and miR-M13. LAT-cluster miRNAs inhibit early viral cytolytic infection and reactivation, suggesting that they may play an active role in the regulation of MDV latency (25). However, the specific miRNAs and mechanisms underlying this process remain unknown.

To explore the functional mechanism of LAT-cluster miRNAs in MDV latency, we screened all nine mature miRNAs derived from the LAT-cluster miRNAs and found that miR-M6-5p significantly inhibited cytolytic replication of MDV *in vitro*. Further studies revealed that the deletion of miR-M6-5p impaired MDV latency, replication, and tumor formation *in vivo*. Importantly, we identified histone demethylase KDM2B as a *bona fide* target of miR-M6-5p, and knockdown of KDM2B increases the level of transcriptionally repressive histone mark H3K27me3 on the important lytic gene pp38 promoter accompanied by suppression of pp38 expression and reduced latent-to-lytic switch in cells latently infected with MDV, indicating that miR-M6-5p plays an important regulatory role in latency of MDV.

## MATERIALS AND METHODS

### Cells and virus

Chicken embryonic fibroblasts (CEFs) were prepared from 9-day-old specific pathogen-free (SPF) embryonated chicken eggs (Vital River, Beijing, China) as previously described (26), and were cultured in M199 medium (Gibco, Carlsbad, CA, USA) supplemented with 10% fetal bovine serum (FBS; Gibco). MDCC-MSB-1 cells were derived from a spleen lymphoma induced by virulent MDV strain BC1 (27) and were cultured in Roswell Park Memorial Institute medium (RPMI 1640; Gibco) supplemented with 10% FBS. DF-1 cells were obtained from the ATCC and cultured in Dulbecco’s modified Eagle medium (DMEM; Gibco) supplemented with 10% FBS. All cells were grown at 37°C with 5% of CO_2_. Very virulent MDV strain RB1B and vaccine strain CVI988/Rispens were preserved in our laboratory.

### Antibodies and reagents

Anti-α-tubulin rabbit antibodies (PM054) were obtained from Medical Biological Laboratories (Japan). Histopaque-1083, Histone deacetylases inhibitor sodium butyrate (NaBu), rabbit polyclonal antibody anti-KDM2B (09–864), horseradish peroxidase (HRP)-conjugated goat anti-rabbit IgG, and anti-chicken IgY antibodies were purchased from Sigma-Aldrich (St. Louis, MO, USA). Antibodies against the histones H3K4me3(39159), H3K9me3(39161), and H3K27me3(39155) were purchased from Active Motif (Carlsbad, CA, USA). Mouse anti-chicken CD4 conjugated with phycoerythrin (PE) antibody (8210-09) was purchased from Southern Biotech (Birmingham, AL, USA). Normal rabbit IgG (2729) was purchased from Cell Signaling Technology (Danvers, MA, USA). Anti-histone H3 rabbit antibodies (ab176842) were obtained from Abcam (Cambridge, MA, USA). Chicken anti-MDV serum was obtained from the National Centre for Veterinary Culture Collection (CVCC, Beijing, China). Lipofectamine 3000 and Lipofectamine RNAiMAX transfection reagents were purchased from Invitrogen (Carlsbad, CA, USA). iTaq Universal SYBR Green Supermix was purchased from Bio-Rad (Hercules, CA, USA). Q5 high-fidelity DNA polymerase was purchased from New England Biolabs (Beverly, MA, USA).

### Oligos

Mimics/inhibitors of miRNAs were synthesized by GenePharma (Shanghai, China), and sense sequences are listed in Table S1. All primers were synthesized by Sangon Biotech (Shanghai, China) and are listed in Table S1.

### Generation of recombinant viruses

Two-step Red-mediated recombination was used to construct miR-M6-5p deletion viruses from the cloned genome of the very virulent strain RB1B, as previously described (28, 29). Briefly, the *galK* cassette containing the flanking homologous sequence of miR-M6-5p was amplified from plasmid pSK-*galK* using the primers M6-galk-forward (F) and M6-galk-reverse (R). The I-SceI-Kan cassette containing a flanking homologous sequence was amplified from pEPkan-S using primers M6-KanS-F and M6-KanS-R. Purified *galK* and I-SceI-Kan cassettes were sequentially electroporated into RB1B BAC-containing *Escherichia coli* (*E. coli*) to replace the two copies of miR-M6-5p by two homologous recombination. In the third recombination step, oligonucleotides that encompass the regions upstream and downstream of the miR-M6-5p (M6-galk-DE-F and M6-galk-DE-R) were used to remove the *galK* gene. Finally, the *Kana* gene was removed by adding arabinose to induce I-SceI expression to generate a seamless deletion in both copies of miR-M6-5p (RB1B BAC-ΔmiR-M6-5p). The deletion mutant virus was reconstituted by transfection of CEF with 2 µg RB1B BAC-ΔmiR-M6-5p using Lipofectamine 3000 according to the manufacturer’s instructions. The deletion of the BAC clone and the recombinant virus were confirmed by PCR and Sanger sequencing using the primers M6-F, M6-R, meq-F, and meq-R. Sequencing results were analyzed using the Snapgene software (www.snapgene.com).

### Animal experiments

One-day-old SPF White Leghorn chickens were purchased from the Beijing Vital River Laboratory (Beijing, China) and randomly divided into three groups. Two groups (*n* = 70 each) were infected by the intra-abdominal route with 1,000 plaque-forming units (PFU) of MDV RB1B and RB1B-ΔmiR-M6-5p in 200 µL of diluent. One group (*n* = 10) was injected with 10^6^ CEFs (200 µL) in the same manner and served as a control. At 4, 7, 10, 14, 17, 21, 28, and 35 dpi, whole blood samples and feather pulp were randomly collected from five birds in each group to measure the MDV genome copy number. At 4 and 7 dpi, 12 birds, and at 10 and 14 dpi, 9 birds from each infected group were humanely euthanized, and spleens were collected to isolate CD4^+^ T cells. At the end of the experiment (35 dpi), all remaining animals were humanely euthanized and tumor lesions were examined. Tumor incidence (%) was calculated as follows: number of chickens that developed tumors/total number ×100.

### Isolation and characterization of CD4^+^ T cells

Spleen tissues collected at different time points after infection were randomly divided into three biological replicates. For example, 12 spleens were divided into 3 groups with 4 spleens per group. Spleens were then squeezed through 30-µm strainers (Miltenyi Biotec, Bergisch Gladbach, Germany) using a disposable syringe plunger, into a 50 mL falcon tube to prepare a single-cell suspension. Histopaque-1083 was used to isolate splenic lymphocytes from a single-cell suspension following the manufacturer’s instructions. Next, CD4^+^ T cells were purified from mononuclear cells using anti-mouse IgG microbeads (Miltenyi Biotec) and PE-conjugated antibodies against CD4 according to the manufacturer’s instructions. The purity of isolated CD4^+^ T cells was examined by flow cytometry (Agilent Technologies, Santa Clara, CA, USA). The data were analyzed using FlowJo Software (version 10.8; BD Life Sciences, San Jose, CA, USA).

### DNA extraction and quantification of MDV

To determine the effect of LAT-miRNAs on MDV growth kinetics, CEFs in 12-well plate were prepared and transfected with 100 nM miRNA controls, miR-M6-3p, miR-M6-5p, miR-M7-3p, miR-M7-5p, miR-M8-3p, miR-M8-5p, miR-M10-3p, miR-M10-5p, miR-M13 mimics, or medium only using Lipofectamine 3000. Twenty-four hours post-transfection, 5 × 10^5^ cells were infected with 1,000 PFU of MDV RB1B or CVI988. Forty-eight hours after infection, DNA was extracted from the cells using a TIANamp Genomic DNA Kit (DP304, TIANGEN, Beijing, China) according to the manufacturer’s instructions. To determine the effect of miR-M6-3p and miR-M6-5p on MDV growth kinetics, transfection of miRNAs and viral infection in CEFs were performed as described above. DNA was extracted at different time points (0, 12, 24, 48, 72, 96, and 120 h) after infection, as aforementioned. To evaluate the effect of miR-M6-5p deletion on viral growth kinetics, CEFs grown in 12-well plates were infected with 1,000 PFU of RB1B or RB1B-ΔmiR-M6-5p. DNA was extracted at different time points (0, 12, 24, 48, 72, 96, and 120 h) after infection, as aforementioned. To determine the effect of KDM2B knockdown on latent MDV infection, MSB1 cells were transfected with 50 nM KDM2B-RNAi 1#, KDM2B-RNAi 2#, or RNAi controls. Twenty-four hours after transfection, the cells were treated with 2.5 mM Nabu (a histone deacetylase inhibitor for the MDV latent-to-lytic switch) or PBS as a solvent control. DNA was extracted at different time points (12, 24, 48, 72, and 96 h) after treatment. To determine the effect of H3K27me3 inhibition on latent MDV infection, MSB1 cells were treated with 5 µM Tazemetostat, 5 µM GSK126, or an equal volume of DMSO as a control. DNA was extracted at different time points (12, 24, 48, 72, and 96 h) after treatment. To determine the effect of miR-M6-5p on the growth kinetics of MDV *in vivo*, DNA was extracted from whole blood samples, the feather follicle epithelium, and isolated CD4^+^ T cells collected during animal experiments, as aforementioned.

MDV copy numbers were quantified by absolute fluorescent quantitative PCR (qPCR) using meq gene primers. Briefly, the concentration of genomic DNA extracted from the above cells or tissues was adjusted to 100 ng/µL, and an equal amount of DNA (1 µL) was subjected to qPCR analysis using iTaq Universal SYBR Green Supermix according to the manufacturer’s instructions. qPCR was performed in triplicates on the same plate using a CFX96 Touch Real-Time PCR Detection System (Bio-Rad). As a standard control, CVI988 BAC DNA was diluted to a series of different copy numbers ranging from 10^8^ to 10^1^ copies/mL. The copy numbers of the MDV genome were calculated using a standard curve and the CT values of the samples.

### Plaque assays

Normal CEF or pre-transfected CEF with 100 nM miRNA controls, miR-M6-3p, or miR-M6-5p, were infected with 200 PFU of MDV RB1B or CVI988. Six days after infection, CEFs were fixed with 4% paraformaldehyde and permeabilized with 0.2% Triton X-100 for 15 min. Next, 1% bovine serum albumin was used for blocking and anti-MDV chicken serum (1:1,000), and HRP-conjugated goat anti-chicken IgY antibodies (1:1000) were used as the primary and secondary antibodies, respectively. Finally, the MDV plaques were visualized using the AEC Peroxidase Substrate Kit (Solarbio, Beijing, China) according to the manufacturer’s instructions. The cells were observed and imaged using a light microscope (Leica, Wetzlar, Germany). A minimum of 50 randomly selected plaques was measured for each virus using ImageJ software (National Institutes of Health, Bethesda, MD, USA). The average plaque size was calculated and presented relative to that of the normal cells. For evaluation of the effect of miR-M6-5p deletion on the size of viral plaques, CEFs were infected with 200 PFU of RB1B or RB1B-ΔmiR-M6-5p. Six days after infection, the size of the plaque was examined as aforementioned.

### RNA isolation and reverse transcription-qPCR (RT-qPCR)

Total RNA was extracted using the RNA FAST200 Total RNA Rapid Extraction Kit (Fastagen Biotech, Shanghai, China) according to the manufacturer’s instructions. To quantify the mRNA expression of MDV ICP4, UL36, pp38, meq, vTR, and cellular KDM2B, cDNA was synthesized using the PrimeScript RT Reagent Kit with gDNA Eraser (TaKaRa, Tokyo, Japan) according to the manufacturer’s protocol. To measure miR-M6-5p expression, cDNA was generated by reverse transcription using miRNA RT-PCR Quantitation Kit (GenePharma). qPCR was performed as previously described. All samples were used in triplicates on the same plate, and the expression of mRNA or miRNA was normalized to that of the glyceraldehyde-3-phosphate dehydrogenase (GAPDH) gene or U6 snRNA, respectively.

### Immunoblotting analysis

To detect the protein expression of KDM2B, MSB1 cells were transfected with miRNA controls, miR-M6-5p mimics, miR-M6-5p inhibitors, KDM2B-RNAi (1–4#) or RNAi controls using Lipofectamine RNAiMAX. Forty-eight hours after transfection, cells were lysed in cell lysis buffer for Western Blotting and IP (Beyotime, Shanghai, China) on ice for 30 min. Lysates were centrifuged at 12,000 rpm for 10 min at 4°C, and proteins in the supernatant were quantified and separated by 8% SDS-PAGE gel. The resolved proteins were then blotted onto polyvinylidene difluoride (PVDF) membranes (Bio-Rad). After blocking with 5% skim milk, the membranes were incubated with anti-KDM2B (1:1,000) and anti-α-Tubulin (1:2,000) antibodies, followed by incubation with HRP-conjugated secondary antibodies. To determine the effect of GSK126 or Tazemetostat on H3K27me3 levels, MSB1 cells were treated with 5 µM Tazemetostat, 5 µM GSK126, or an equal volume of DMSO as a control. At 48 h after treatment, cell lysates were harvested for Western blotting using anti-H3K27me3 (1:1,000) or anti-Histone H3 antibodies (1:5,000), followed by detection with HRP-conjugated secondary antibodies. Reactive blots were visualized using an enhanced chemiluminescence (ECL) kit (Thermo Fisher Scientific), according to the manufacturer’s instructions.

### Luciferase reporter gene assays

To construct the luciferase reporter gene, 200 bp flanking sequences of predicted miRNA binding sites in KDM2B, EP300, RBBP4, DHX38, and METTL6 3′UTR were first amplified by PCR using Q5 high-fidelity DNA polymerase and then cloned into the psiCHECK-2 reporter vector by homologous recombination using the pEASY-Basic Seamless Cloning and Assembly Kit (TransGen Biotech, Beijing, China), resulting in psiCHECK-2-target-3'UTR. Specific multisite mutations were introduced at miRNA-binding sites by PCR-based site-specific mutagenesis, resulting in psiCHECK-2-target-3′UTR-mut.

For the luciferase assay, DF-1 cells were seeded in 24-well plates and cultured overnight, followed by transfection with 50 ng of psiCHECK-2, psiCHECK-2-target-3′UTR, or psiCHECK-2-target-3′UTR-mut and 100 nM miRNA controls or miR-M6-5p mimics using Lipofectamine 3000. Forty-eight hours post-transfection, luciferase activity was measured using the Dual-Luciferase Reporter Assay System (Promega, Madison, WI, USA) according to the manufacturer’s instructions. Firefly luciferase was used as an internal control to normalize the Renilla luciferase activity.

### Knockdown of KDM2B by RNAi

To knock down KDM2B in MSB1 cells, four siRNAs were designed and synthesized by GenePharma. The sense sequences of the KDM2B-siRNAs and RNAi control are listed in Table S1. Next, 50 nM KDM2B-siRNAs (1–4#) or RNAi controls were transfected into MSB1 cells in 12-well plates using Lipofectamine RNAiMAX. Forty-eight hours after transfection, the cells were harvested for Western blotting analysis.

### Chromatin immunoprecipitation assay

KDM2B-RNAi 1#, KDM2B-RNAi 2#, RNAi controls, miR-M6-5p, or miRNA controls were transfected into MSB1 cells in a 10 cm plate using Lipofectamine RNAiMAX. Chromatin immunoprecipitation (ChIP) assays were performed with Pierce Magnetic ChIP Kit (Thermo Fisher) according to the manufacturer’s instructions. The extracted chromatins were incubated with 5 µL anti-histone H3K4me3, anti-histone H3K9me3, or anti-histone H3K27me3 antibodies for immunoprecipitation. For each ChIP assay, the same amount of normal rabbit IgG was used as a negative control. The precipitated DNA was analyzed by qPCR as described above. Ten percent of the total DNA was used as input DNA. The enrichment of histone modifications in specific genomic regions was calculated as a percentage of immunoprecipitated DNA relative to the input DNA. All ChIP data are representative of three independent experiments.

### Cell viability assay

MSB1 cells at a density of 5 × 10^4^ cells/mL were plated in 96-well plates and treated with 5 µM Tazemetostat, 5 µM GSK126, or an equal volume of DMSO as a control. At 48 h after treatment, cell viability was detected using Enhanced Cell Counting Kit-8 (Beyotime) according to the manufacturer’s instructions. The absorbance at 450  nm was measured using a microplate reader (Perkin-Elmer; Waltham, MA, USA).

### Statistical analyses

Statistical analyses were performed using GraphPad Prism software (version 8.0; La Jolla, CA, USA). Data derived from at least three independent experiments with three replicates are presented as the mean ± SD. The significant differences in tumor incidence between different groups were determined and analyzed using Fisher’s exact test. Significant differences in viral load, gene expression, plaque area, luciferase activity, and histone methylation enrichment between the treated and control groups were determined and analyzed using the Mann-Whitney U test or analysis of variance (ANOVA).

## RESULTS

### Multiple miRNAs of the LAT cluster are associated with restricting MDV replication in CEFs

**Fig 1:**
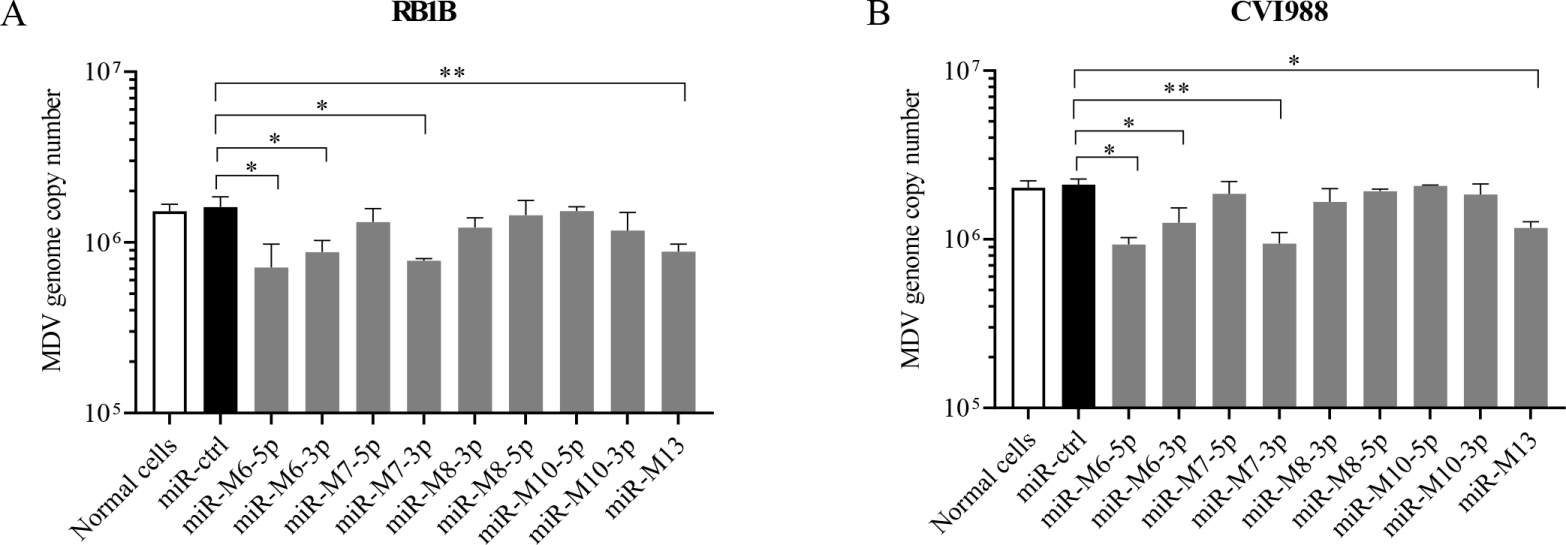
Screening of miRNA candidates in the LAT-cluster miRNA involved in viral cytolytic replication. The CEFs were prepared and transfected with 100 nM miRNA controls, miR-M6-3p, miR-M6-5p, miR-M7-3p, miR-M7-5p, miR-M8-3p, miR-M8-5p, miR-M10-3p, miR-M10-5p, miR-M13 mimics, or medium only using Lipofectamine 3000. At 24 h post-transfection, 5 × 10^5^ cells were infected with 1,000 PFU of the very virulent MDV strain RB1B (**A**) or the vaccine strain CVI988 (**B**). At 48 h post-transfection, DNA was extracted and the meq gene was detected by qPCR to quantify the MDV genome. Data are representative of three independent experiments with three replicates and presented as means ± SD. ^**^P < 0.01; ^*^P < 0.05; Mann-Whitney U test.

Previous studies have shown that LAT cluster miRNAs restrict early cytolytic replication of MDV (25). To identify the specific miRNAs involved in viral replication, we screened nine mature miRNAs derived from the LAT cluster. Nine miRNA mimics were synthesized and transfected into CEFs followed by MDV infection to examine their effects on the lytic replication of MDV. Among the nine miRNA candidates, miR-M6-5p, miR-M6-3p, miR-M7-3p, and miR-M13 overexpression significantly reduced the genome copy number of the very virulent MDV strain RB1B (Fig. 1A) and vaccine strain CVI988 (Fig. 1B) compared to that of miRNA controls. These data suggest that multiple MDV-encoded miRNAs in the LAT cluster may be involved in restricting viral replication in CEFs.

### miR-M6-5p restricts MDV replication in CEFs

**Fig 2:**
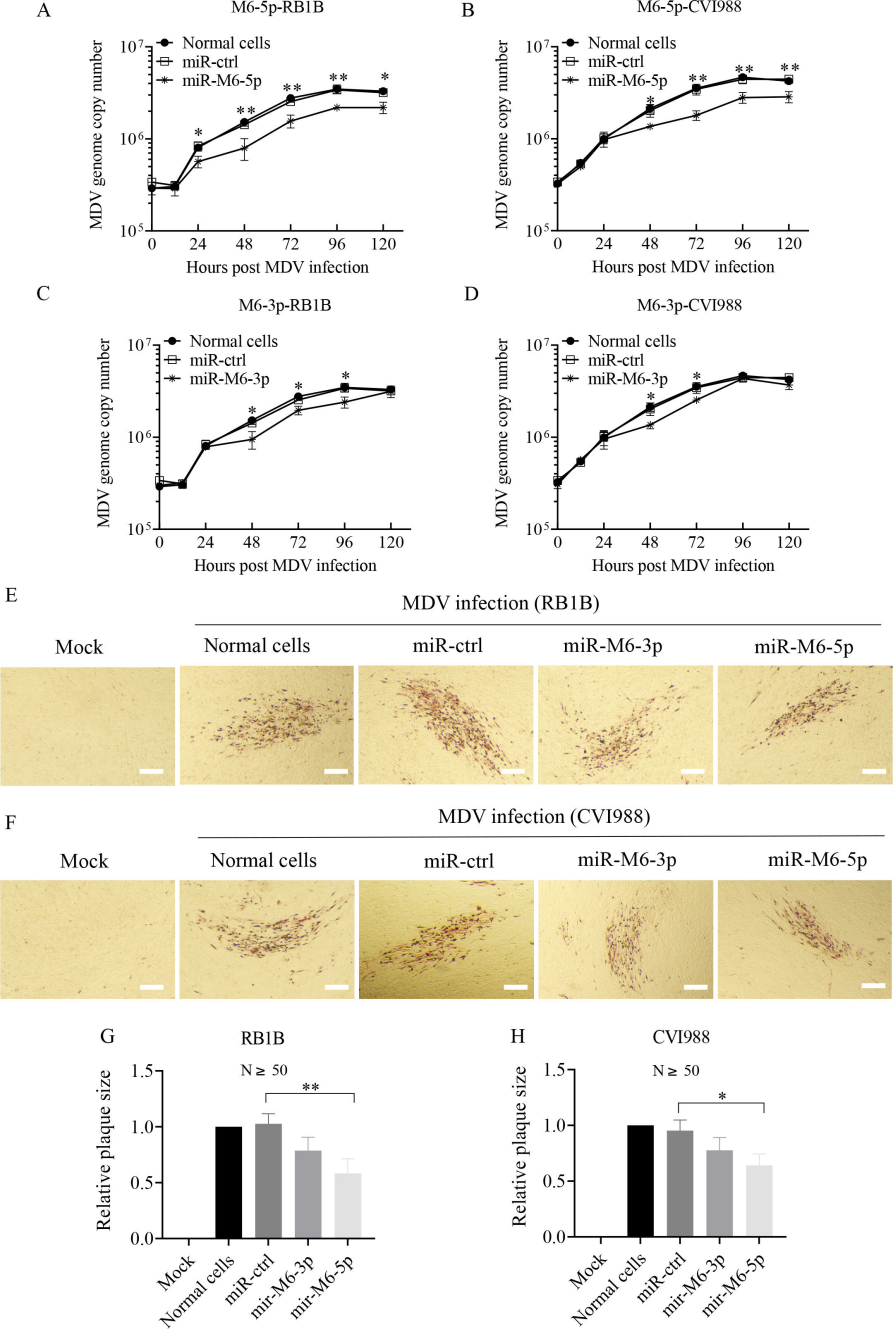
Transfection of CEFs with miR-M6-5p inhibits MDV replication. (A to D) Analysis of the effect of miR-M6-5p or miR-M6-3p on the growth kinetics of MDV *in vitro*. CEFs were prepared and transfected with 100 nM miRNA controls, miR-M6-5p (**A and B**), miR-M6-3p (**C and D**), or medium only. At 24 h post-transfection, 5 × 10^5^ cells were infected with 1,000 PFU of the RB1B strain (**A and C**) or CVI988 strain (**B and D**). At the indicated times post-infection, DNA was extracted and the meq gene was detected by qPCR to quantify the MDV genome. (E to H) Analysis of the effect of miR-M6-5p or miR-M6-3p on the plaque size of MDV. CEFs were prepared and transfected with 100 nM miRNA controls, miR-M6-5p, miR-M6-3p, or medium only. At 24 h post-transfection, cells were infected with 200 PFU of the RB1B (**E**) or CVI988 strain (**F**). Six days after infection, plaque assays were performed. For each virus, the area of at least 50 plaques was measured. The relative plaque size was calculated and shown in panel G (RB1B) and panel H (CVI988). The scale bar in the picture represents 50 µm. Data are representative of three independent experiments with three replicates and presented as mean ± SD. ^**^P < 0.01; ^*^P < 0.05; Mann-Whitney U test.

Among the LAT cluster miRNAs that inhibit MDV replication, we focused on miR-M6, including miR-M6-5p and miR-M6-3p, which showed relatively strong inhibitory effects on viral replication. To further determine the role of miR-M6-5p and miR-M6-3p in MDV replication, the effect of miR-M6-5p and miR-M6-3p on MDV growth kinetics and plaque size was examined. The genome copy numbers for MDV RB1B and CVI988 infection in CEFs transfected with miR-M6-5p or miR-M6-3p were significantly lower than those in the controls (Fig. 2A through D). Plaque sizes of RB1B and CVI988 were significantly reduced in cells overexpressing miR-M6-5p rather than in cells overexpressing miR-M6-3p (Fig. 2E through H). These data clearly show that compared with miR-M6-3p, miR-M6-5p has a stronger inhibitory effect on MDV replication.

**Fig 3:**
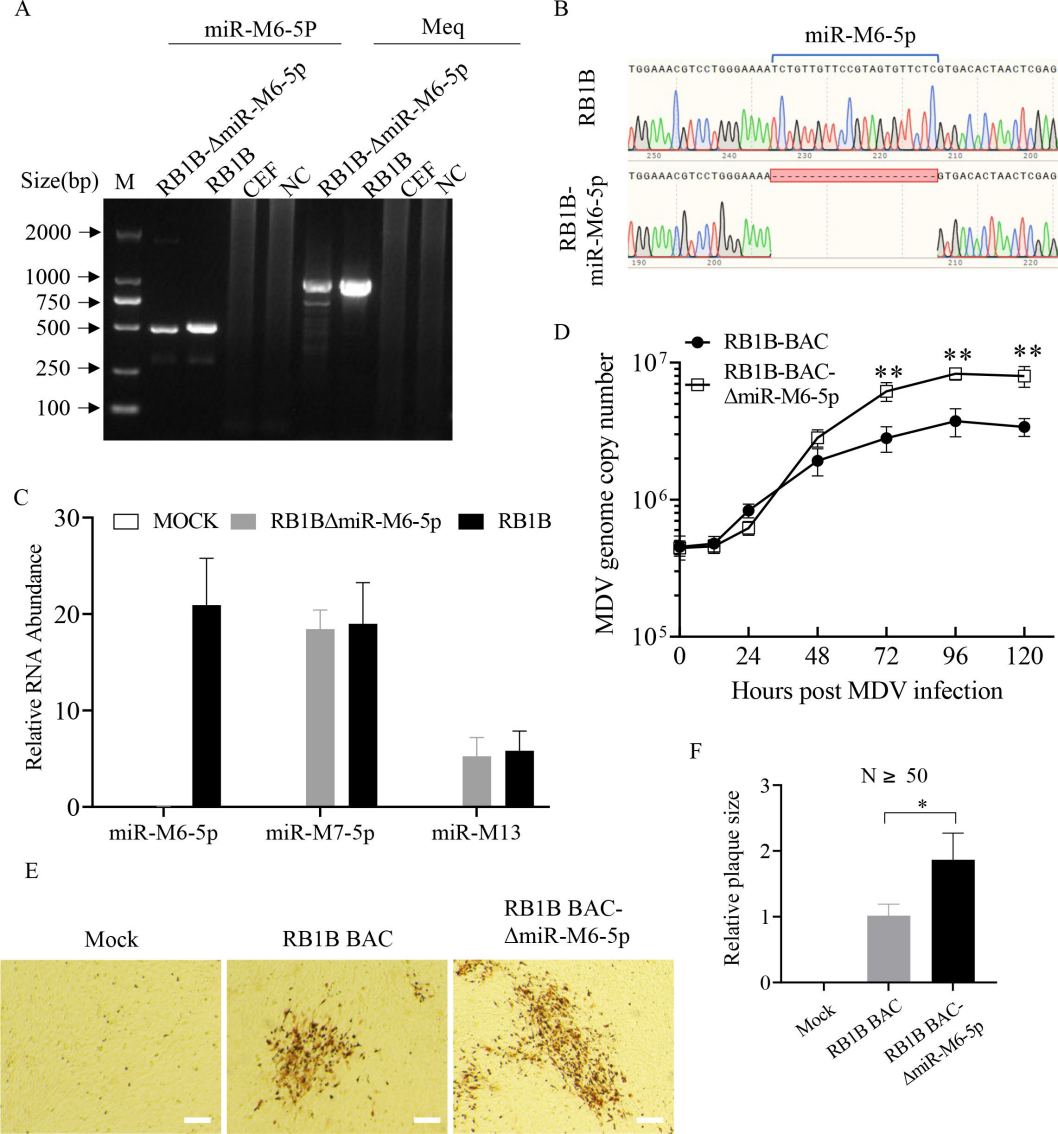
*In vitro* characterization of miR-M6-5p deletion viruses. (A to D) CEFs were mock infected or infected with 1,000 PFU of the RB1B or RB1B-ΔmiR-M6-5p virus. (**A**) At 4 days after infection, DNA was extracted and used to amplify the miR-M6-5p region and the meq gene to confirm the miR-M6-5p deletion viruses by PCR. (**B**) PCR amplification products of miR-M6-5p regions from wild type and deletion virus were sequenced and aligned (**C**) At 48 h post-infection, total RNA was extracted and qRT-PCR was performed to detect miR-M6-5p, miR-M7-5p, and miR-M13 transcripts. The expression of U6 was used as an internal control, and miRNA expression levels were normalized to viral genome levels. (**D**) DNA was extracted at different time points (0, 12, 24, 48, 72, 96, and 120 h) after infection and used to quantify viral genomes by qPCR. (**E and F**) Analysis of plaque size of miR-M6-5p deletion viruses. CEFs were mock-infected or infected with 200 PFU of RB1B BAC or RB1B -ΔmiR-M6-5p virus. At 6 days post-infection, plaque assays were performed (**E**). For each virus, the area of at least 50 plaques was measured. The relative size of the plaque was calculated and shown in panel F. The scale bar in the picture represents 50 µm. Data are representative of three independent experiments with three replicates and are presented as means ± SD. ^**^P < 0.01; ^*^P < 0.05; Mann-Whitney U test.

To further investigate the role of miR-M6-5p in MDV replication and pathogenesis, a recombinant virus lacking miR-M6-5p sequences (RB1B-ΔmiR-M6-5p) was generated using a bacterial artiﬁcial chromosome (BAC) of the RB1B strain (30) (Fig. S1). Deletion of miR-M6-5p was confirmed by PCR and sequencing analyses. The PCR result showed that the product of the miR-M6-5p gene region amplified from RB1B-ΔmiR-M6-5p was 22 bp less than that amplified from the parental RB1B BAC viruses (Fig. 3A). Sequencing of the PCR products confirmed that this reduced 22 bp was the result of miR-M6-5p deletion (Fig. 3B). As a control, PCR results for the meq gene of the deleted and parental strains were identical (Fig. 3A). Consistently, the expression of miR-M6-5p was undetectable in CEFs infected with RB1B-ΔmiR-M6-5p viruses. Meanwhile, the deletion did not affect the expression of neighboring viral miR-M7-5p and miR-M13 (Fig. 3C). We then determined the growth kinetics and plaque size of the different MDVs in CEFs. The results showed that the deletion of miR-M6-5p markedly increased the copy number of the MDV RB1B genome in CEFs compared to that of the parental virus (Fig. 3D). Plaque size assay further revealed that the deletion of miR-M6-5p increased plaque area in infected CEFs (Fig. 3E and F). Taken together, these results suggest that miR-M6-5p is a key restriction factor involved in the lytic replication of MDV.

### Deletion of miR-M6-5p impairs latent MDV infection in CD4^+^ T cells *in vivo*

**Fig 4:**
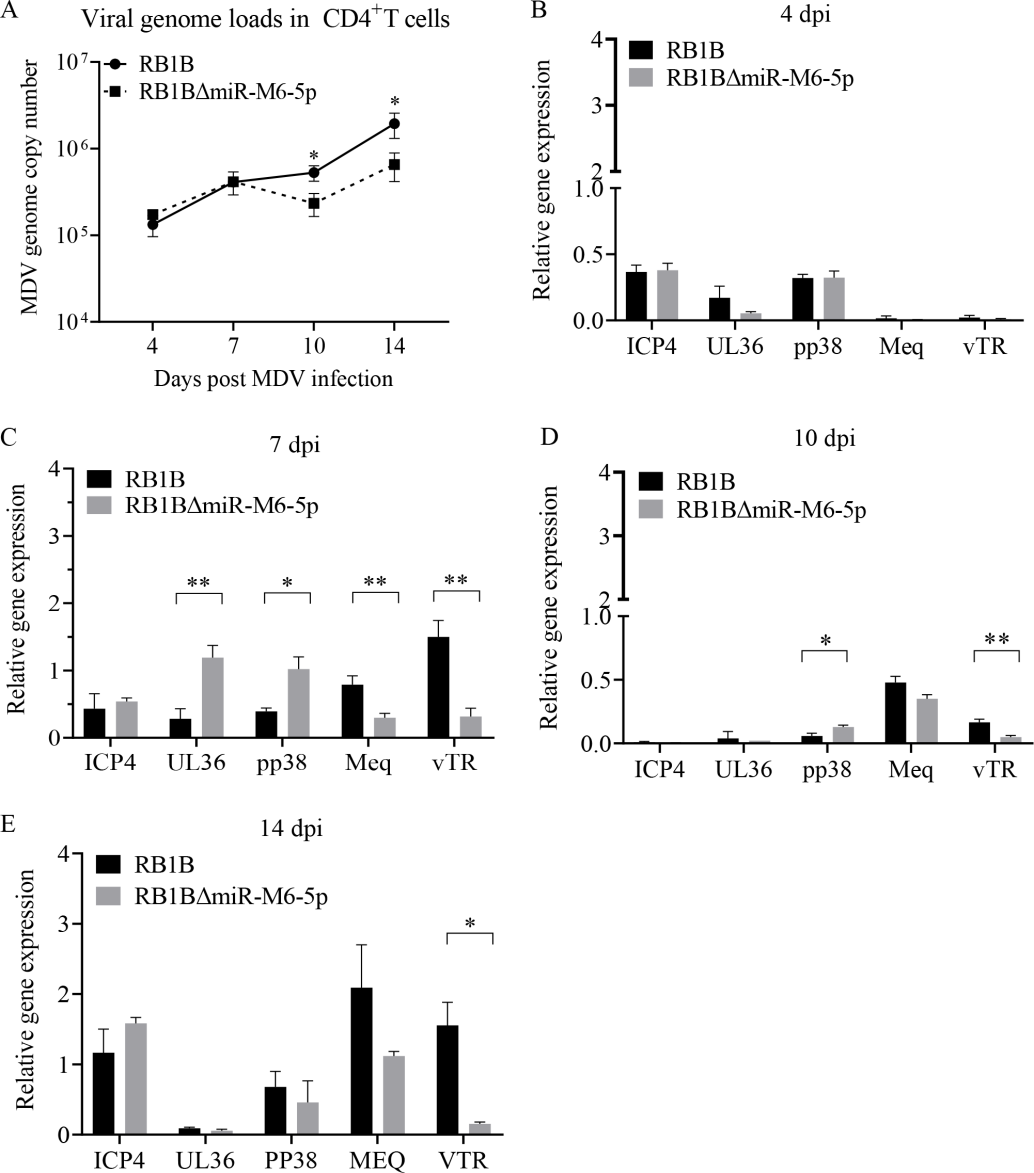
Effect of miR-M6-5p deletion on MDV latency in CD4^+^ T cells *in vivo*. (**A**) Growth kinetics of miR-M6-5p deletion viruses in CD4^+^ T cells *in vivo*. DNA was extracted from CD4^+^ T cells isolated from the spleen at 4, 7, 10, and 14 dpi, and qPCR was performed to determine the MDV genome copy number. (**B-E**) Effect of miR-M6-5p deletion on multiple viral gene expression in CD4^+^ T cells infected with MDV. At 4 (B), 7 (C), 10 (D), and 14 (E) dpi, RNA was extracted from CD4^+^ T cells isolated from the spleen. The mRNA expressions of ICP4, UL36, pp38, meq, and vTR were detected by qRT-PCR. The expression of GAPDH was used as an internal control. Relative gene expression was calculated using CFX Maestro Software. Data are representative of at least three replicates and are presented as means ± SD. ^**^P < 0.01; ^*^P < 0.05; Mann-Whitney U test.

Suppression of viral lytic replication and non-production of infectious virions are common characteristics of herpesvirus latency (31). The fact that miR-M6-5p can inhibit MDV replication suggests that miR-M6-5p may be involved in the regulation of latent MDV infection. To test this hypothesis, one-day-old chickens were inoculated with 1,000 PFU of RB1B-ΔmiR-M6-5p or the parental RB1B BAC virus. At different time points after infection, CD4^+^ T cells, where MDV primarily establishes latent infection, were sorted from the spleen, and the purity of isolated CD4^+^ T cells was examined by flow cytometry (Fig. S2). Subsequently, we examined the MDV genome copy number, expression of viral lytic transcripts (ICP4, UL36, and pp38), and latency-associated transcripts (meq and vTR) in isolated CD4^+^ T cells. At 4 dpi (lytic infection), the viral genome load and the expression of several viral genes were not significantly different between the two viruses (Fig. 4A and B). However, at 7 dpi (establishment of latency), the expression of UL36 and pp38 increased, while the expression of meq and vTR decreased in CD4^+^ T cells infected with RB1B-ΔmiR-M6-5p virus (Fig. 4C); at 10 dpi (latency), deletion of miR-M6-5p markedly reduced viral genome copy number, increased the expression of the pp38 gene and repressed the expression of vTR compared to that of parental virus (Fig. 4A and D); at 14 dpi (reactivation phase), viral loads and the expression of vTR decreased in CD4^+^ T cells infected with deletion strain (Fig. 4E and A). Collectively, these results illustrate that the miR-M6-5p deletion reduces the MDV genome copy number accompanied by increased lytic transcripts and reduced latency-associated transcripts during latency *in vivo*, suggesting that the miR-M6-5p deletion impairs latent infection of MDV *in vivo*.

### Deletion of miR-M6-5p results in impaired second cytolytic viral infection and complete replication *in vivo*

**Fig 5:**
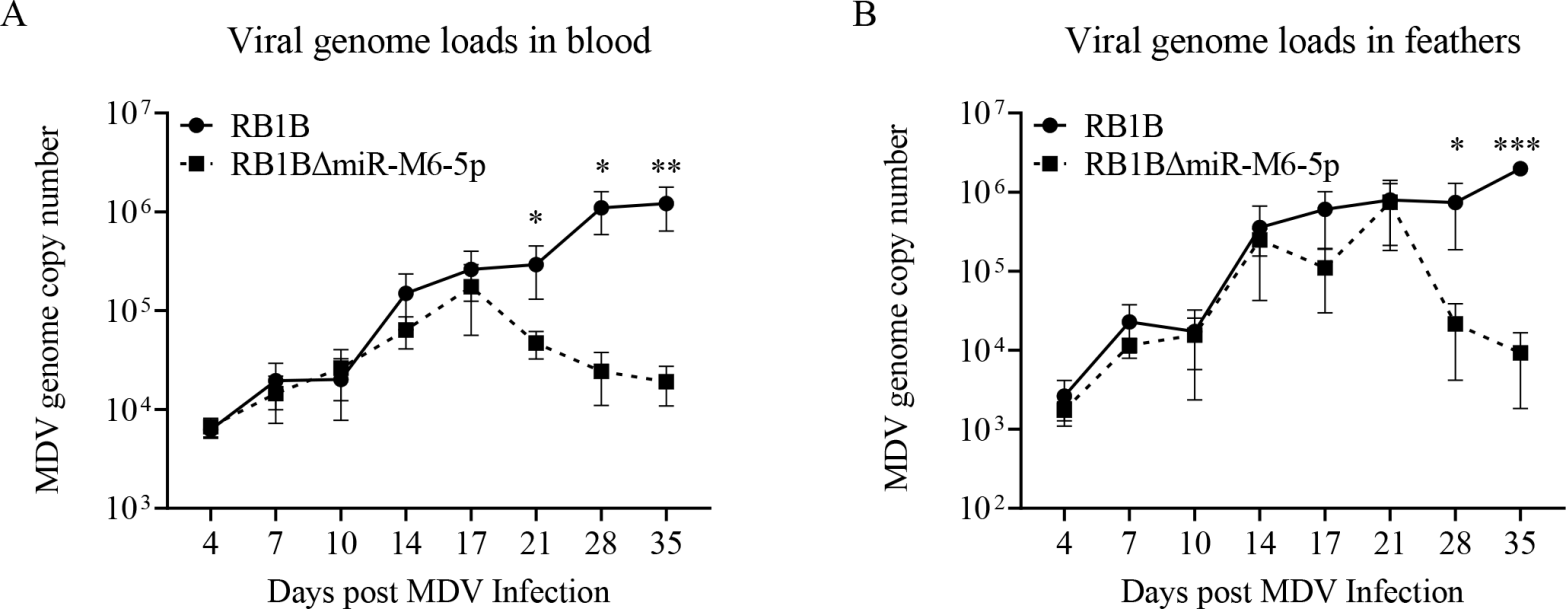
Viral genome loads in the blood and feathers of inoculated chickens. DNA was extracted from blood (**A**) or feathers (**B**) of chickens inoculated with the RB1B or RB1B-ΔmiR-M6-5p viruses on the indicated days after infection. The viral genomes were measured by qPCR. Data are representative of at least three replicates and are presented as means ± SD. ^***^P < 0.001; ^**^P < 0.01; ^*^P < 0.05; Mann-Whitney U test.

To further assess the effect of miR-M6-5p on MDV proliferation *in vivo*, we performed qPCR to determine the MDV genome copy number in whole blood and feather follicle epithelium at specific time points. As shown in Fig. 5, starting at 21 dpi, the viral load in the blood of chickens infected with the RB1B-ΔmiR-M6-5p virus was significantly lower than that of chickens infected with the parental virus (Fig. 5A). Furthermore, the deletion of miR-M6-5p resulted in a significant reduction in the viral load in the feather follicle epithelium compared to the parental virus at 28 and 35 dpi (Fig. 5B). These data demonstrate that miR-M6-5p plays an important role in viral replication during the second cytolytic infection phase of MDV.

### Deletion of miR-M6-5p suppressed tumor development

To investigate whether miR-M6-5p affects MDV-induced oncogenicity, we recorded the incidence rate of lymphomas in chickens infected with RB1B-ΔmiR-M6-5p and the parent RB1B at the end of animal experiments (35 dpi). Compared with the 50% tumor incidence in the visceral organs of chickens infected with the parent RB1B virus, only 21.4% of birds infected with RB1B-ΔmiR-M6-5p virus developed MD lymphomas during the 35-day experimental period (Table 1). The findings that the RB1B-ΔmiR-M6-5p virus decreased tumor incidence, albeit not statistically significant, suggest that miR-M6-5p could promote lymphoma development during 5 weeks.

**TABLE 1:** Summary of visceral tumor incidences

Group	No. of chickens	Tumor incidence
Negative control	9	0/9 (0%)
RB1B	14	7/14 (50%)^*a*^
RB1BΔmiR-M6-5p	14	3/14 (21.4%)^*a*^

^*a*^ Indicates no significant difference (*P* > 0.05; Fisher’s exact test) between the two groups labeled.

### Histone demethylase KDM2B gene is a direct target of miR-M6-5p

**Fig 6:**
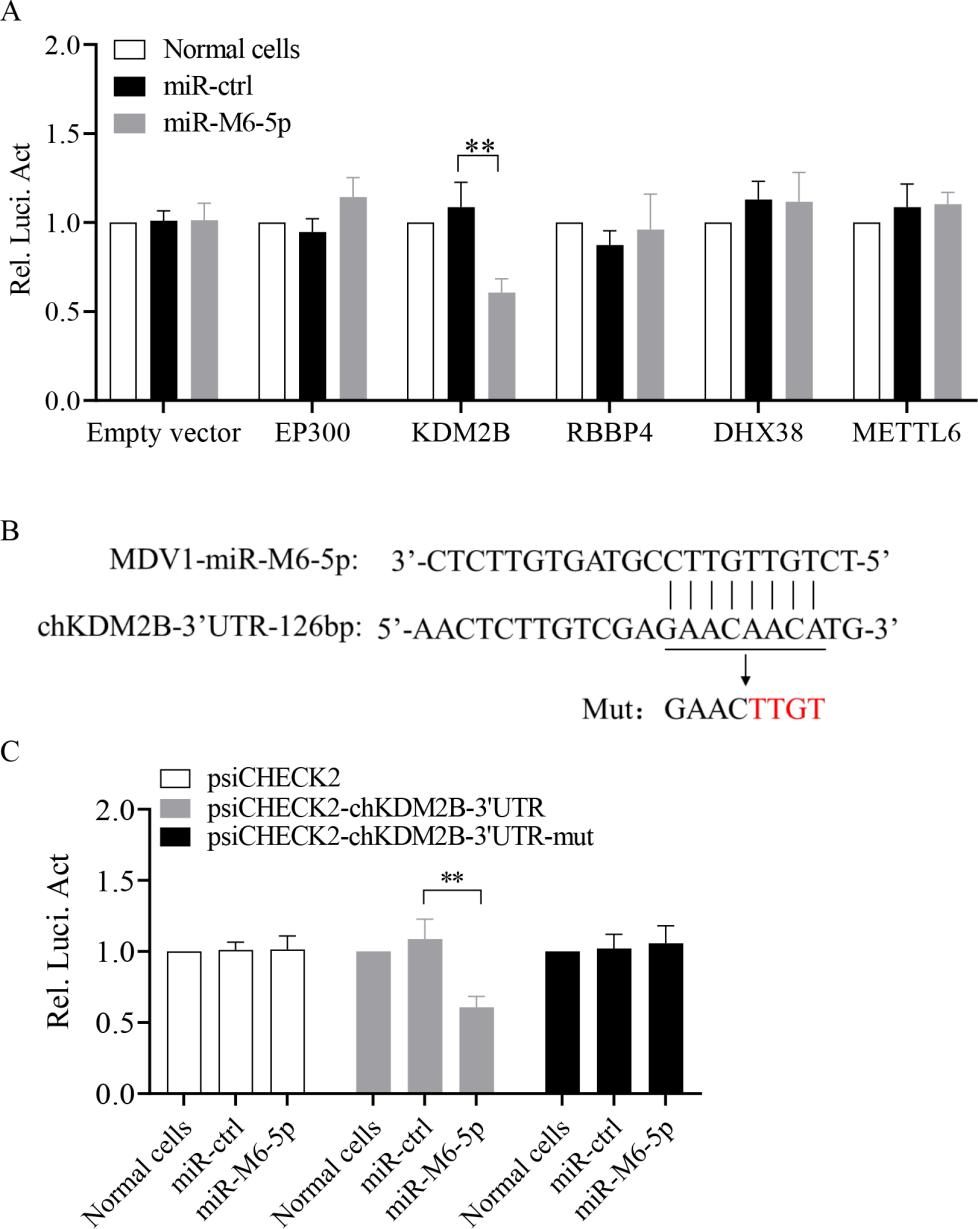
miR-M6-5p directly targets cellular gene KDM2B. (**A**) Screening of target genes of miR-M6-5p. DF-1 cells were cotransfected with miR-M6-5p, controls, and luciferase reporter constructs. At 48 h post-transfection, cells were lysed, and luciferase activity was measured using Dual-luciferase assays. (**B**) Diagram of the predicted target site for miR-M6-5p in the KDM2B gene. The predicted miR-M6-5p binding sites in KDM2B 3′UTR are underlined and mutated as indicated by the arrow. (**C**) Overexpression of miR-M6-5p in DF-1 cells reduced the expression of KDM2B but not its mutant. Dual-luciferase assays were performed as described above in panel A. Relative levels of luciferase activity (Rel Luc Act) were calculated as follows: (luciferase activity of cells transfected with reporter plasmids together with miR-M6-5p mimics)/(luciferase activity of cells cotransfected with reporter plasmid and miRNA controls). Data are representative of three independent experiments with three replicates and presented as means ± SD. ^**^P < 0.01; Mann-Whitney U test.

As miR-M6-5p is involved in the regulation of latent MDV infection and pathogenesis, it is intriguing to identify the targets of miR-M6-5p to explore the underlying molecular mechanisms. In a previous study, potential target genes of miR-M6-5p were identified by photoactivatable ribonucleoside-enhanced crosslinking and immunoprecipitation (PAR-CLIP) analysis (32). Based on this study, some of the potential target genes involved in the regulation of gene transcription were selected for further validation, such as chicken gene EP300, KDM2B, RBBP4, DHX38, and METTL6. Luciferase reporter plasmids were constructed by inserting the individual predicted target site of these genes, and then cotransfected with miRNAs into DF-1 cells followed by dual-luciferase reporter gene assays. The results showed that among these candidate targets, luciferase activity was significantly reduced in DF-1 cells transfected with miR-M6-5p and reporter plasmid of KDM2B (Fig. 6A). To determine whether miR-M6-5p specifically inhibits KDM2B, we also constructed luciferase reporter plasmid (psiCHECK-2-KDM2B-3′UTR-mut) by insertion of mutated sequences (Fig. 6B), then performed dual-luciferase reporter gene assays. As shown in Fig. 6C, miR-M6-5p transfection significantly inhibited Renilla luciferase reporter activity compared to miRNA control. This inhibition was completely rescued by mutating the target site in the luciferase reporter plasmid. These results demonstrate that miR-M6-5p directly targets binding sites in the 3′UTR of KDM2B.

**Fig 7:**
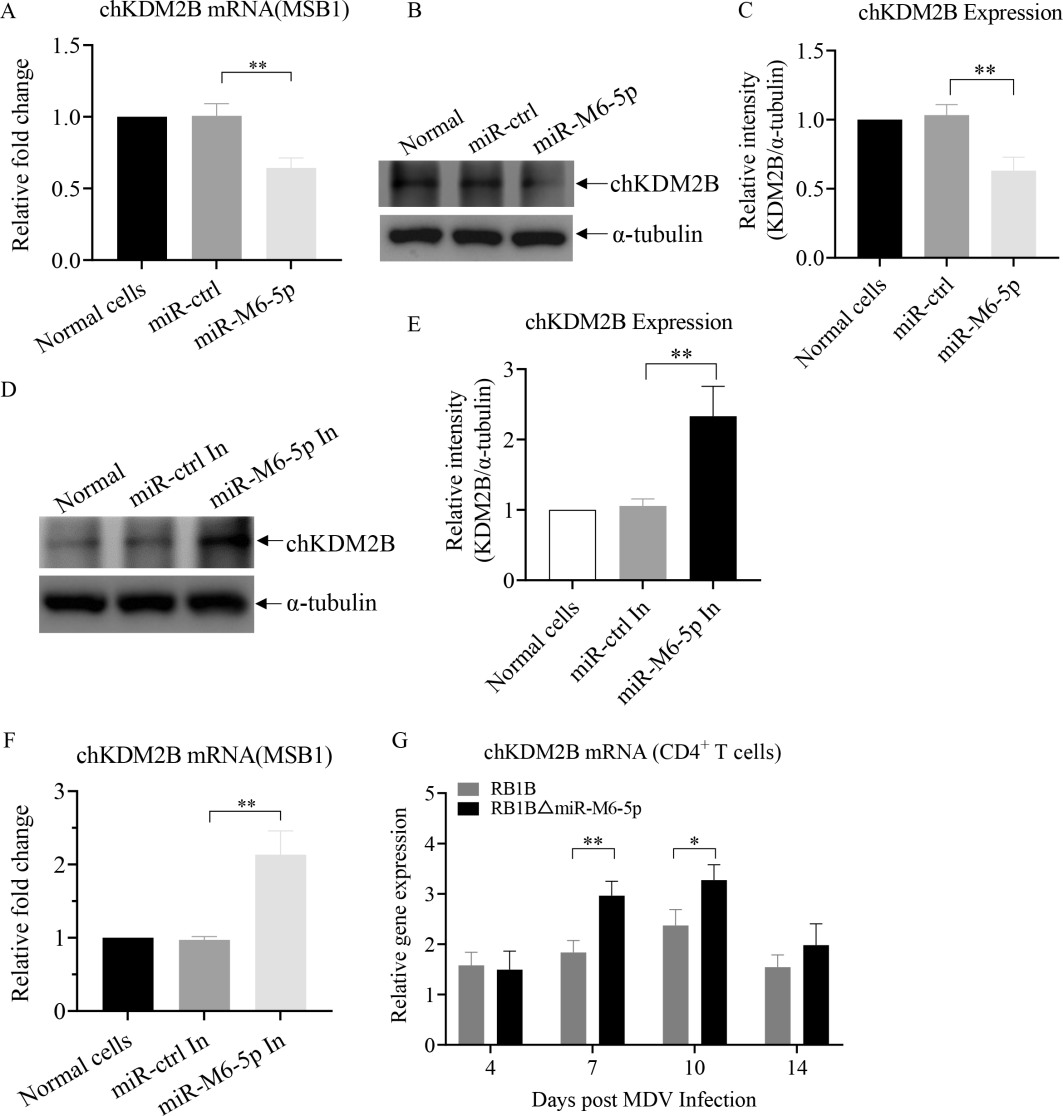
miR-M6-5p inhibits KDM2B expression during MDV latency. (A to C) Effect of miR-M6-5p overexpression on KDM2B expression in MSB1 cells. MSB1 cells were transfected with 100 nM miRNA controls, miR-M6-5p, or medium only. (**A**) At 24 h post-transfection, qRT-PCR was performed to examine KDM2B transcripts. (**B and C**) At 48 h post-transfection, MSB1 cell lysates were prepared and KDM2B protein expression was examined by Western blotting using anti-KDM2B antibodies (**B**), and (**C**) the band densities of KDM2B in panel B were quantitated by densitometry. (D to F) Effect of endogenous miR-M6-5p knockdown on KDM2B expression in MSB1 cells. MSB1 cells were transfected with 100 nM miRNA inhibitor controls (miR-Inh ctrl), miR-M6-5p Inh, or medium alone. At 24 h post-transfection, qRT-PCR was performed to examine KDM2B transcripts (**F**). At 48 h, the expression of the KDM2B protein was examined by Western blotting using anti-KDM2B antibodies (**D**), and (**E**) the band densities of KDM2B in panel D were quantitated by densitometry. (**G**) Effect of deletion of miR-M6-5p on the expression of KDM2B mRNA in CD4^+^ T cells *in vivo*. At indicated days post-infection, RNA was extracted from CD4^+^ T cells isolated from the spleen. KDM2B mRNA expressions were detected by qRT-PCR. GAPDH was used as an internal control for qRT-PCR. Relative levels of gene expression were calculated as follows: (KDM2B mRNA expression in transfected cells)/ (mRNA expression of that in normal cell controls). Endogenous α-tubulin expression was examined as an internal control for Western blotting. The relative levels of KDM2B were calculated as follows: band density of KDM2B/that of α-tubulin. Data are representative of three independent experiments with three replicates and are presented as means ± SD. ^**^P < 0.01; ^*^P < 0.05; Mann-Whitney U test.

As an epigenetic factor, KDM2B regulates target gene expression by changing histone methylation modification (33, 34). We assumed that miR-M6-5p might modulate the latent MDV epigenome by targeting KDM2B, thus regulating viral gene expression and latent infection. To further elucidate the relationship between miR-M6-5p and KDM2B, we measured the expression levels of KDM2B mRNA and proteins under overexpression or knockdown of miR-M6-5p. KDM2B mRNA and protein expression levels were significantly decreased by overexpression of miR-M6-5p in MSB1 cells (T-lymphoid cell line latently infected with MDV) (Fig. 7A through C). Conversely, the knockdown of endogenous miR-M6-5p using specific inhibitors significantly increased both the expression of KDM2B mRNA and protein in MSB1 cells (Fig. 7D through F). Consistently, the level of KDM2B mRNA was increased in CD4^+^ T cells *in vivo* infected with RB1B-ΔmiR-M6-5p virus at 7 and 10 dpi compared with that of the parental virus (Fig. 7G). These data show that miR-M6-5p directly targets KDM2B during MDV latent infection.

### KDM2B is critical in maintaining MDV latency

**Fig 8:**
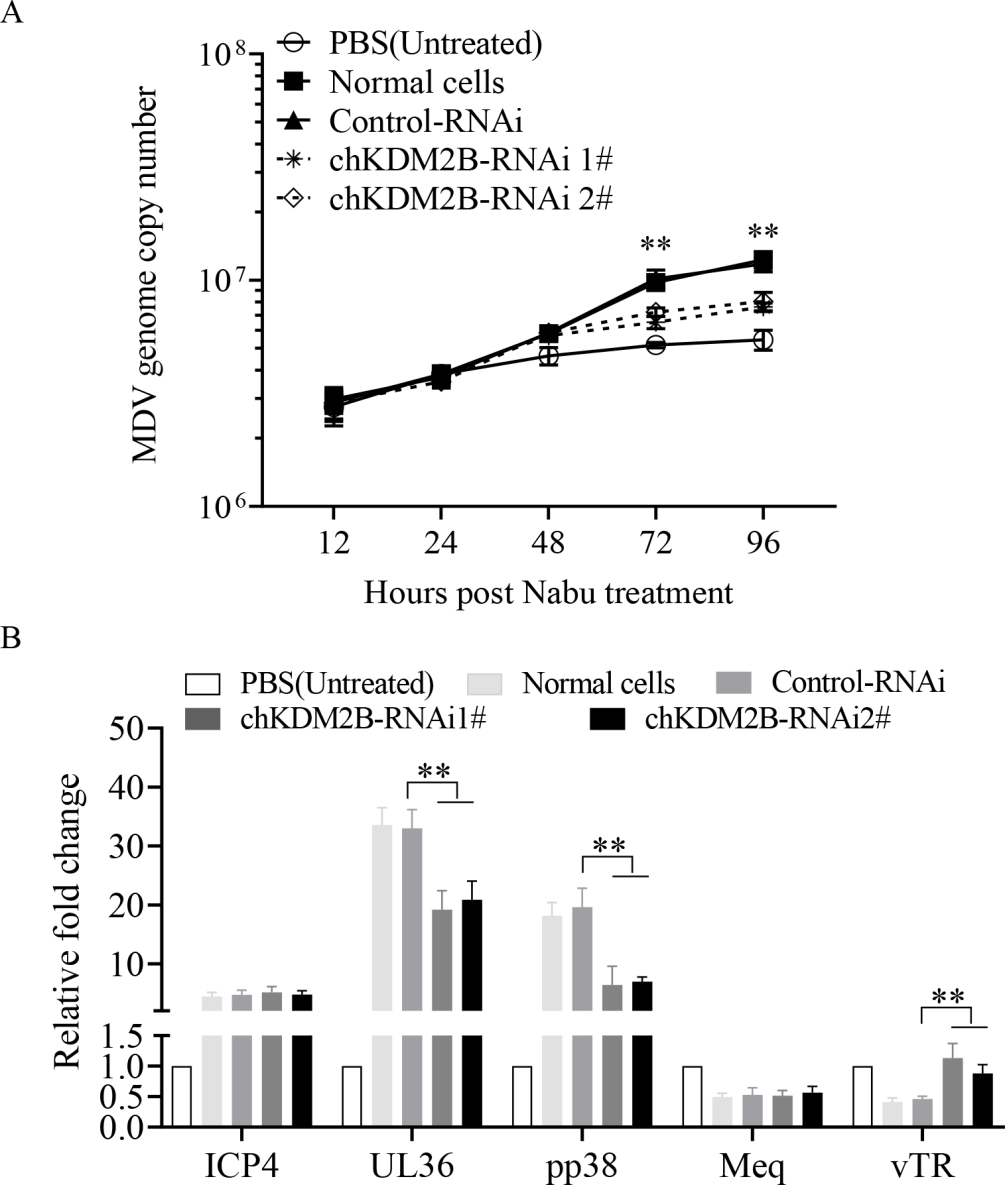
Effects of endogenous KDM2B knockdown on latent MDV infection in MSB1 cells. MSB1 cells were transfected with KDM2B-RNAi 1#, KDM2B-RNAi 2#, RNAi controls, or medium only. At 24 h post-transfection, cells were treated with 2.5 mM Nabu or the corresponding solvent (PBS). (**A**) At different time points (12, 24, 48, 72, and 96 h) after treatment, DNA was extracted and qPCR was performed to measure the MDV genome copy number. (**B**) At 48 h after treatment, mRNA expressions of ICP4, UL36, pp38, meq, and vTR were detected by qRT-PCR. GAPDH was used as an internal control. Relative levels of gene expression were calculated as follows: (mRNA expression of ICP4, UL36, pp38, meq or vTR in treated cells)/ (mRNA expression of normal cell controls). Data are representative of three independent experiments with three replicates and are presented as means ± SD. ^**^P < 0.01; ^*^P < 0.05; one-way analysis of variance (ANOVA).

Having found that miR-M6-5p is involved in latent MDV infection and that KDM2B is a direct target of miR-M6-5p, we further evaluated the importance of KDM2B in MDV latency. First, the efficiency of the KDM2B RNAi constructs was evaluated in MSB1 cells. As shown in Fig. S3, transfection of MSB1 cells with KDM2B RNAi 1# and 2# effectively lowered the cellular levels of KDM2B. Then, KDM2B knockdown MSB1 cells were treated with sodium butyrate to trigger MDV reactivation from latency (10). The results showed that the MDV genome copy number was significantly lower in MSB1 cells transfected with KDM2B RNAi than in MSB1 cells transfected with RNAi controls, although MDV replication dramatically increased in both cells treated with sodium butyrate (Fig. 8A). Furthermore, KDM2B knockdown using RNAi in sodium butyrate-treated MSB1 cells suppressed the expression of the viral lytic genes UL36 and pp38 and promoted the expression of the latency-associated gene vTR compared to RNAi controls (Fig. 8B). These results suggest that KDM2B plays a critical role in maintaining MDV latency by reducing or delaying reactivation.

### Overexpression of miR-M6-5p or the knockdown of KDM2B increases the level of the repressive histone marks H3K27me3 on the pp38 promoter in MSB1 cells

**Fig 9:**
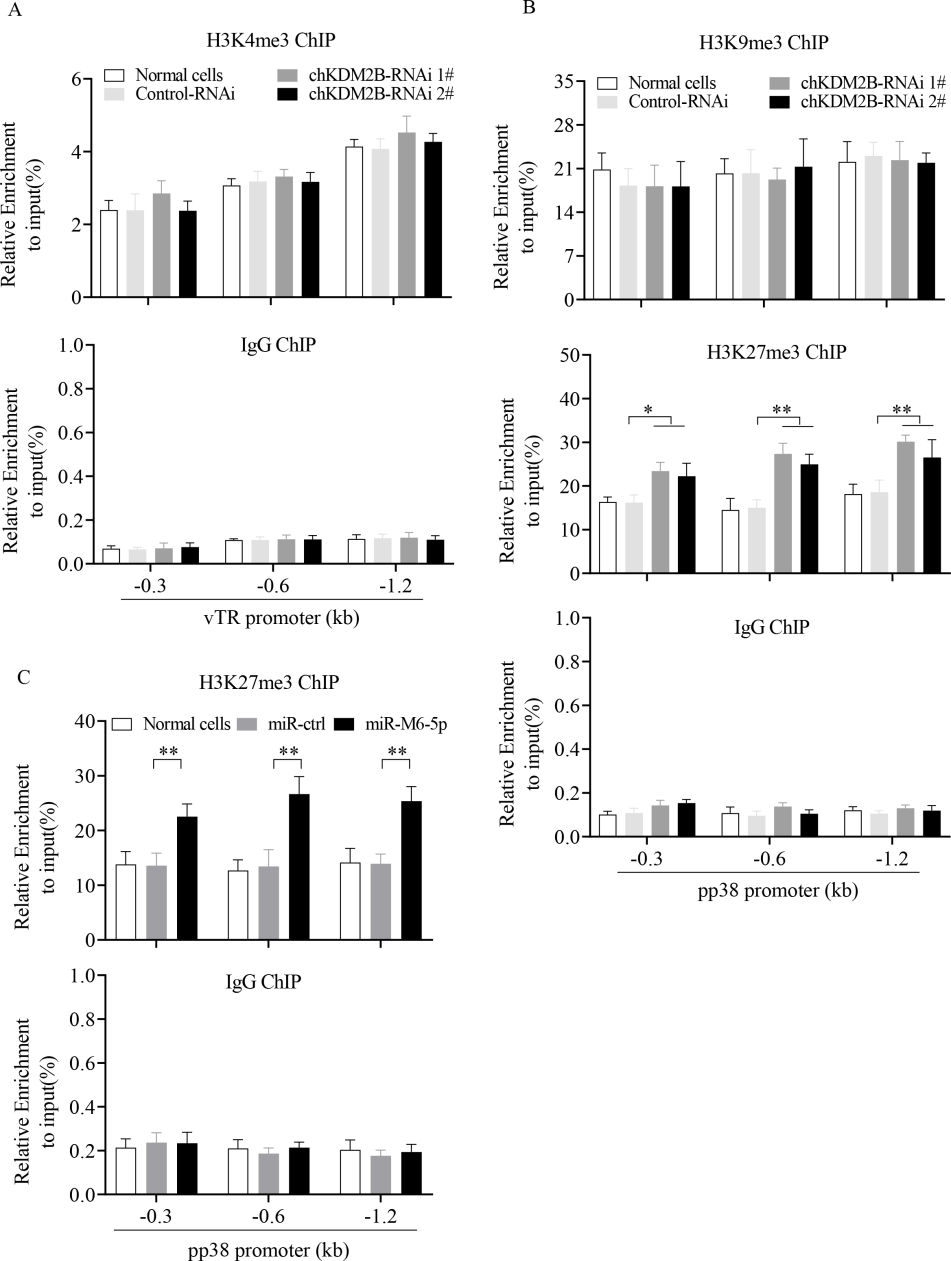
Effect of miR-M6-5p overexpression or endogenous KDM2B knockdown on the change in histone marks at sites within the pp38 and VTR loci in MSB1 cells. MSB1 cells were transfected with KDM2B-RNAi 1#, KDM2B-RNAi 2#, RNAi controls, miR-M6-5p, miRNA controls, or medium only. At 48 h post-transfection, ChIP was performed for (**A**) H3K4me3, (**B**) H3K9me3, and (**B and C**) H3K27me3. Rabbit IgG ChIP was used as a negative control. Enrichment of histone modifications in different upstream regions of vTR (**A**) and pp38 (**B and C**) were detected by qPCR. Relative enrichment was calculated as a percentage of immunoprecipitated DNA compared to the input DNA. Data are representative of three independent experiments with three replicates and presented as means ± SD. ^**^P < 0.01; ^*^P < 0.05; one-way analysis of variance (ANOVA) (**B**) or Mann-Whitney U test (**C**).

Previous studies have shown that the transcriptionally repressive marks H3K27me3 and H3K9me3 are present on the promoter of the lytic gene pp38, and the transcriptional activation mark H3K4me3 is present on the promoter of latency-associated genes vTR and meq in cells latently infected with MDV, suggesting that histone modifications play an important role in maintaining the latent genome of MDV (11). Therefore, we aimed to determine whether KDM2B regulates the expression of the expression of pp38 and vTR by affecting the enrichment of histone methylation marks in the promoters of pp38 and vTR in MSB1 cells. To this end, histone mark ChIP analysis was performed on the viral genes pp38 and vTR in KDM2B—knockdown MSB1 cells. The results showed that KDM2B knockdown did not affect H3K4me3 levels in the vTR promoter (Fig. 9A) or H3K9me3 levels in the pp38 promoter (Fig. 9B), whereas it significantly increased the level of H3K27me3 in the pp38 promoter (Fig. 9B). We also examined the effects of miR-M6-5p overexpression on H3K27me3 levels at the pp38 promoter in MSB1 cells. Consistently, miR-M6-5p overexpression significantly increased the level of H3K27me3 on the pp38 promoter (Fig. 9C). These results indicate that miR-M6-5p increases repressive histone marks H3K27me3 modification on the pp38 promoter *via* targeting KDM2B.

### Inhibition of histone H3K27me3 impairs MDV latency in MSB1 cells

**Fig 10:**
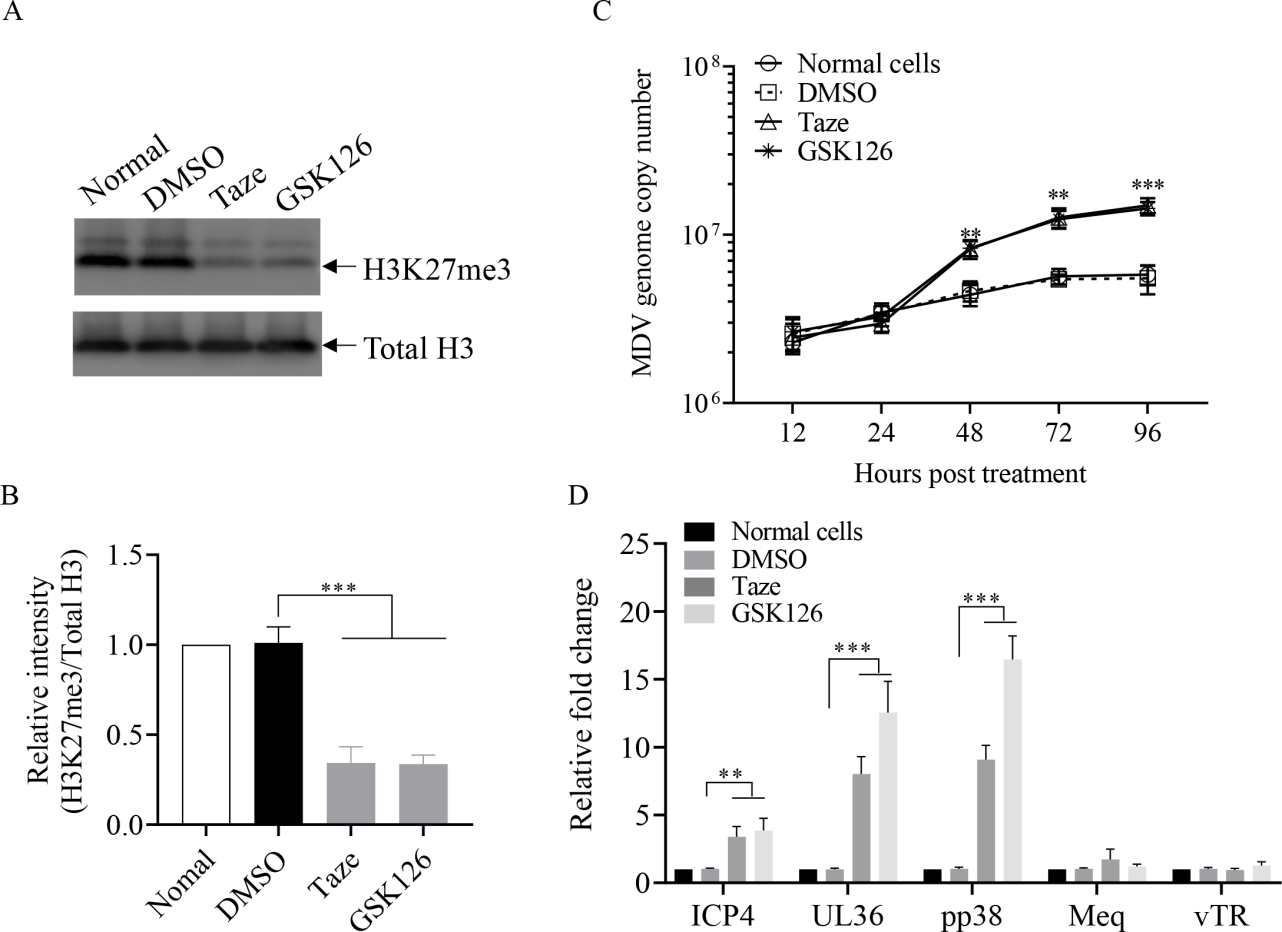
Effects of H3K27me3 inhibitors on latent MDV infection in MSB1 cells. MSB1 cells were treated with 5 µM Tazemetostat (Taze), 5 µM GSK126, or an equal volume of dimethyl sulfoxide (DMSO) as a control. (**A**) At 48 h after treatment, MSB1 cell lysates were prepared and H3K27me3 protein expression was examined by Western blotting using anti-H3K27me3 antibodies, and (**B**) the band densities of H3K27me3 in panel B were quantitated by densitometry. Endogenous total histone H3 expression was examined as an internal control for Western blotting. The relative levels of H3K27me3 were calculated as follows: band density of H3K27me3/that of total H3. (**C**) At different time points (12, 24, 48, 72, and 96 h) after treatment, DNA was extracted and qPCR was performed to measure the MDV genome copy number. (**D**) At 48 h after treatment, mRNA expressions of ICP4, UL36, pp38, meq, and vTR were detected by qRT-PCR. GAPDH was used as an internal control. Relative levels of gene expression were calculated as follows: (mRNA expression of ICP4, UL36, pp38, meq or vTR in treated cells)/ (mRNA expression of that in normal cell controls). Data are representative of three independent experiments with three replicates and presented as means ± SD. ***P < 0.001; **P < 0.01; one-way analysis of variance (ANOVA).

As miR-M6-5p increases the transcriptionally repressive marks the H3K27me3 on viral genome and maintains latent infection by targeting KDM2B, histone H3K27me3 modification could possibly be involved in regulating latent infection of MDV. Therefore, we treated MSB cells with a nontoxic dose of GSK126 or Tazemetostat (Fig. S4), two inhibitors of H3K27me3 methyltransferase EZH2, to determine the effect of inhibition of H3K27me3 on latent MDV infection in MSB1 cells. As expected, treatment of MSB1 cells with the H3K27me3 methyltransferase inhibitor significantly reduced H3K27me3 levels compared to that of controls (Fig. 10A and B). Furthermore, inhibition of H3K27me3 in MSB1 cells by GSK126 or Tazemetostat significantly increased the copy number of MDV genome (Fig. 10C) and expression of viral lytic transcripts ICP4, UL36, and pp38, compared with the controls (Fig. 10D). These results suggest that the histone marker H3K27me3 was associated with the maintenance of MDV latency in MSB1 cells.

Altogether, these results show that miR-M6-5p facilitates MDV latency by suppressing the expression of the viral lytic gene pp38 *via* targeting histone demethylase KDM2B accompanied by increased transcriptionally repressive histone marks H3K27me3 at the pp38 promoter, which can contribute to the second viral cytolytic replication and tumor formation *in vivo*.

## DISCUSSION

**Fig 11:**
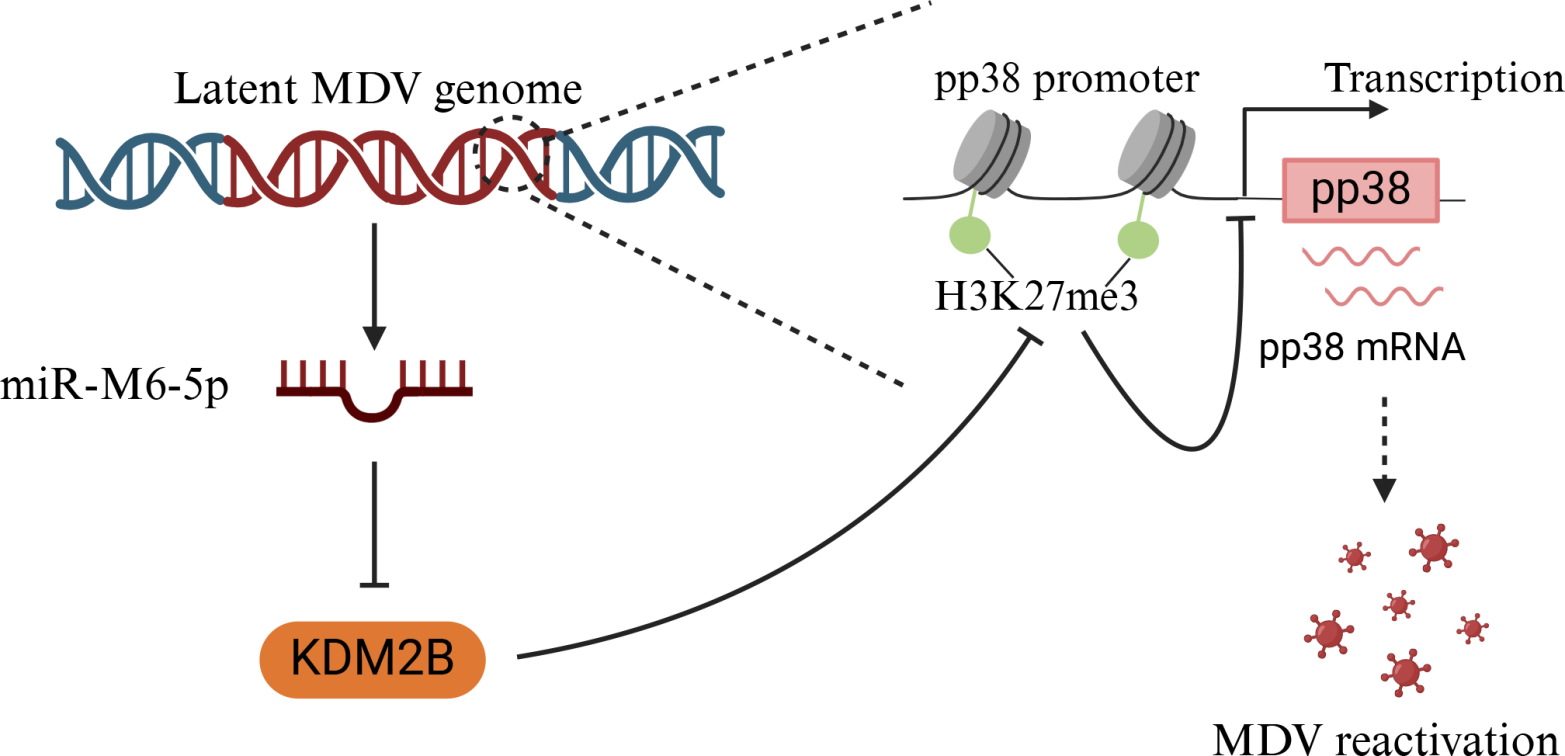
Working model for miR-M6-5p regulating MDV latency by targeting KDM2B. miR-M6-5p facilitates MDV latency by suppressing the expression of the viral lytic gene pp38 *via* targeting histone demethylase KDM2B accompanied by increased transcriptionally repressive histone marks H3K27me3 at the pp38 promoter. Image created using BioRender.com, with permission.

The highly pathogenic and oncogenic avian alphaherpesvirus MDV naturally infects chickens and endangers poultry worldwide with considerable economic implications (35). Similar to other herpesviruses, MDV establishes a latent infection in the host, allowing viruses to persist throughout their lifetime (36). Although latent infection is an important component of the MDV life cycle, the molecular mechanisms underlying MDV latency remain unclear. In the present study, we found that miR-M6-5p facilitates latent viral infection, late replication, and tumor formation *in vivo*. Our findings also revealed that by directly targeting the histone demethylase KDM2B, miR-M6-5p represses the expression of the MDV lytic gene pp38 and enhances the expression of the latency-associated gene vTR. Furthermore, we unveiled the underlying mechanism by which miR-M6-5p suppresses pp38 expression by increasing repressive epigenetic modification of H3K27me3 at the pp38 promoter *via* targeting KDM2B in latently infected MDV cells (Fig. 11). Therefore, we demonstrated that miR-M6-5p serves as a positive regulator of MDV latency and lymphoma development, highlighting the critical role of virus-encoded miRNAs in MD pathogenesis.

Latency is a signature trait developed by herpesviruses during their long-term coevolution with their hosts. MDV establishes a latent infection, primarily in CD4^+^ T cells (37). During latent infection, MDV expresses only a few transcripts, including the viral oncogene meq (38), LAT (5), viral telomerase RNA (vTR) (39, 40), and MDV-encoded miRNAs (41, 42), which are presumed to be involved in the regulation of MDV latency. MDV can integrate into the host genome to maintain its latent genome. As a template for telomerase, the vTR is involved in the integration of MDV into the host genome and is believed to facilitate MDV latency (39). As a transcriptional regulator encoded by MDV, the meq homodimer can bind to the promoter of the key lytic genes pp38 and pp24 and inhibit their transcriptional activity (43), which is considered to promote the latency of MDV. Several MDV-encoded miRNAs directly target viral genes and inhibit their expression; for example, miR-M5-3p and miR-M1-5p target and downregulate the immediate-early (IE) gene ICP22 (44), miR-M4-5p targets and downregulates the viral assembly associated genes UL28 and UL32 (45), and miR-M7-5p targets and downregulates the lytic genes ICP4 and ICP27 (46). This suggests that these miRNAs may be involved in establishing MDV latency by suppressing the expression of lytic replication-related genes. However, the specific roles and mechanisms of action of these molecules in MDV latency remain unclear due to the lack of suitable cellular models for studying MDV latency *in vitro*. In this study, by isolating CD4^+^ T cells from chickens at different time points after infection, we found that miR-M6-5p facilitates MDV latency *in vivo* (Fig. 4). Our data showed a significant decrease in viral growth rate and almost undetectable expression of lytic genes in isolated CD4^+^ T cells at 10 dpi (Fig. 4), suggesting that MDV established a latent infection, whereas the significant increase in viral growth rate and expression of lytic genes at 14 dpi (Fig. 4) indicates that MDV reactivation was induced, which is consistent with the previously reported replication cycle of MDV in chickens (37). To our knowledge, this is the first study to examine the regulation of MDV latency by viral molecules in CD4^+^ T cells *in vivo*.

Although we found that miR-M6-5p restricted MDV productive replication *in vitro* (Fig. 2 and 3), animal experiments showed that deletion of miR-M6-5p did not affect early cytolytic infection of MDV (initial 7 dpi), but suppressed viral replication at 21–35 dpi in feathers and blood (Fig. 5). Therefore, we speculate that in addition to being influenced by the complex environment *in vivo*, miR-M6-5p may play a critical role in the maintenance of MDV latency rather than in the establishment of latency. Moreover, we found that deletion of miR-M6-5p significantly reduced the incidence of MDV-induced tumors (Table 1). Previous studies have shown that the deletion of telomeric repeats, which are necessary for the integration of MDV into the host genome, also reduces viral loads at 21 dpi and tumor incidence (13). Taken together, these results suggest that MDV latency plays an important role in viral pathogenesis. We also noted that a previous study by Yang et al. constructed a very virulent MDV strain Md5 mutants lacking miR-M6-5p and showed higher replication levels in CEFs (47), which is consistent with our findings (Fig. 3). However, our results are not in line with the findings of this study; there was no significant difference in the viral load in the spleen at 5, 14, and 63 dpi *in vivo*, and there was little difference in the tumor incidence between Md5 mutants lacking miR-M6-5p (22.2%) and the parental viruses (11.1%) (47). We believe that the differences in our results may be due to the use of different MDV clones to test the effects of miR-M6-5p on MDV replication and pathogenesis *in vivo*.

MiRNAs bind to target sequences in mRNAs, known as miRNA recognition elements (MREs), *via* imperfect base-pairing interactions (48). The length of the MREs is approximately 6–7 nt, indicating that a miRNA could have multiple potential target genes. The target genes of MDV-encoded miR-M4-5p have been identified to include not only the viral genes UL28 and UL32 (45) but also many host genes such as WWOX (49), LTBP1 (50), hnRNPAB (51), and SOCS1 (52). In this study, we identified for the first time the target gene of miR-M6-5p, KDM2B. Meanwhile, we found that deletion of miR-M6-5p promoted the expression of MDV pp38 and UL36 and repressed the expression of vTR and meq (Fig. 4), while KDM2B was not involved in the regulation of meq expression (Fig. 8B). Considering the important role of meq in latent infection and tumorigenesis, miR-M6-5p may play a role by regulating meq expression through other targets; however, further studies are needed to confirm this idea.

Polycomb repressive complexes (PRCs) include two major classes, namely PRC1 and PRC2, that regulate target gene expression through histone modifications. Histone demethylase KDM2B, a key component of noncanonical PRC1 (PRC1.1), mediates the enrichment of H3K27me3 (53, 54). However, we found that KDM2B downregulated the transcriptionally repressive methylation mark H3K27me3 at the promoter of the viral lytic gene pp38 and promoted pp38 expression in latent MDV-infected cells (Fig. 8B and 9B). This suggests a dual effect of KDM2B on H3K27me3 modification, and the mechanism by which KDM2B reduces H3K27me3 modification needs to be further investigated. pp38 is required for the lytic replication of MDV in B cells and the first viral protein expressed in FFE after reactivation (55, 56). A recent study found that activation of pp38 expression in MDV latently infected cells using the CRISPRa Tet-On system, which up-regulated the expression of key viral lytic genes ICP4, pp14, and gB, increased the production of infectious viral particles, and triggered the switch from latent to lytic infection (57). Thus, KDM2B regulates MDV latency by epigenetically altering the expression of pp38. Furthermore, we found that KDM2B repressed the expression of the latency-associated gene, vTR, although it did not affect the level of the transcriptionally active marker, H3K4me3, at the vTR promoter. Studies have shown that in addition to histone methylation modifications, KDM2B can also regulate gene expression by recognizing unmethylated CpG islands in the DNA sequence, recruiting the PRC1 protein with E3 ubiquitin ligase activity, and modifying the histone H2 ubiquitination (58). KDM2B also interacts with BRG1, the core catalytic subunit of the chromatin remodeling complex, increasing chromatin accessibility and recruiting RNA polymerase II to regulate downstream gene expression (59). Therefore, KDM2B could regulate the expression of vTR independently of histone demethylase activity, a mechanism that requires further study.

Collectively, our investigations revealed the mechanism by which virus-encoded miRNAs play critical roles in latent MDV infection and pathogenesis. This will broaden our understanding of the underlying pathogenic mechanisms of MDV and will provide a rationale for the development of novel strategies for the prevention and control of MD.

## Data Availability

The data that support the findings of this study are available from the corresponding author, upon reasonable request.

## SUPPLEMENTAL MATERIAL

Supplemental figures jvi.02007-24-s0001.pdf Figures S1 to S4.

Table S1 jvi.02007-24-s0002.docx Sequences of oligonucleotides used in this study.

ASM does not own the copyrights to Supplemental Material that may be linked to, or accessed through, an article. The authors have granted ASM a non-exclusive, world-wide license to publish the Supplemental Material files. Please contact the corresponding author directly for reuse.
