# Supplementary material for: Marek’s disease virus-encoded microRNA-M6-5p facilitates viral latent infection by targeting histone demethylase KDM2B
Source: J Virol. 2025 Jan 22;99(2):e02007-24. doi: 10.1128/jvi.02007-24 (PMC11853111; doi:10.1128/jvi.02007-24)
Supplement: Supplemental figures — Figures S1 to S4. [file jvi.02007-24-s0001.pdf]

S1 Fig

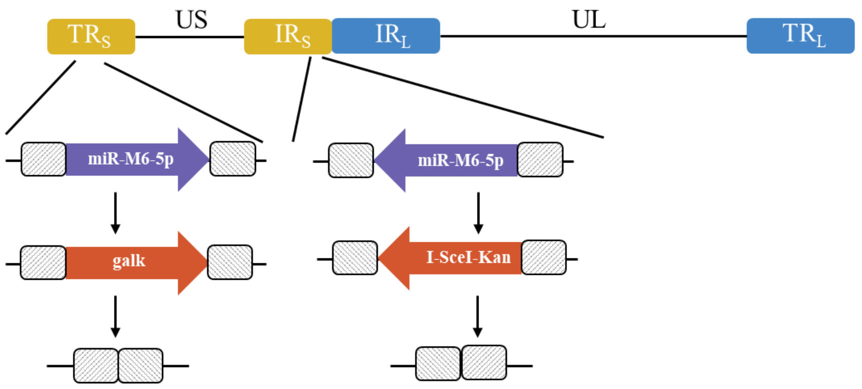

S1 Fig Schematic diagram of the deletion miR-M6-5p. The RB1B genome consists of a unique long region (UL) and a unique short region (US) flanked by a long terminal repeat and a long internal repeat (TRL and IRL, respectively) and by a short terminal repeat and a short internal repeat (TRS and IRS, respectively). Two copies of miR-M6-5p are located in the TRS and IRS regions of the genome, respectively. Deletion of both copies of miR-M6-5p by two-step Red-mediated recombination is illustrated in the figure. Shaded boxes represent genes region flanking miR-M6-5p.

S2 Fig

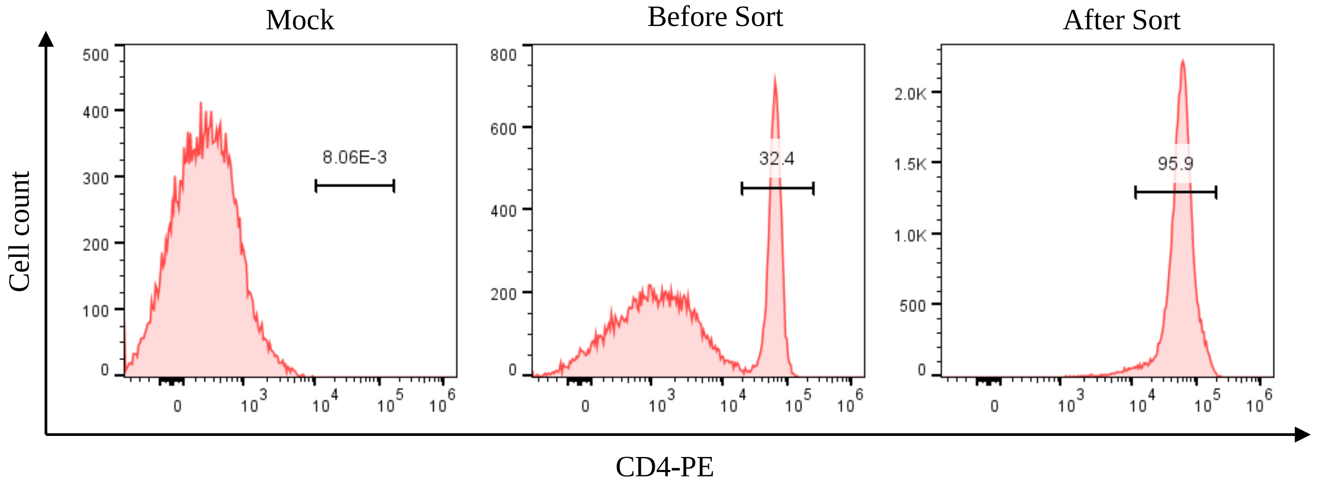

S2 Fig Examination of purity of the isolated CD4$^+$ T cells from chicken spleen. CD4$^+$ T cells were sorted from the spleen using magnetic beads and PE-conjugated CD4 antibodies at the indicated days post infection. The purity of the obtained cells was examined using flow cytometry.

S3 Fig

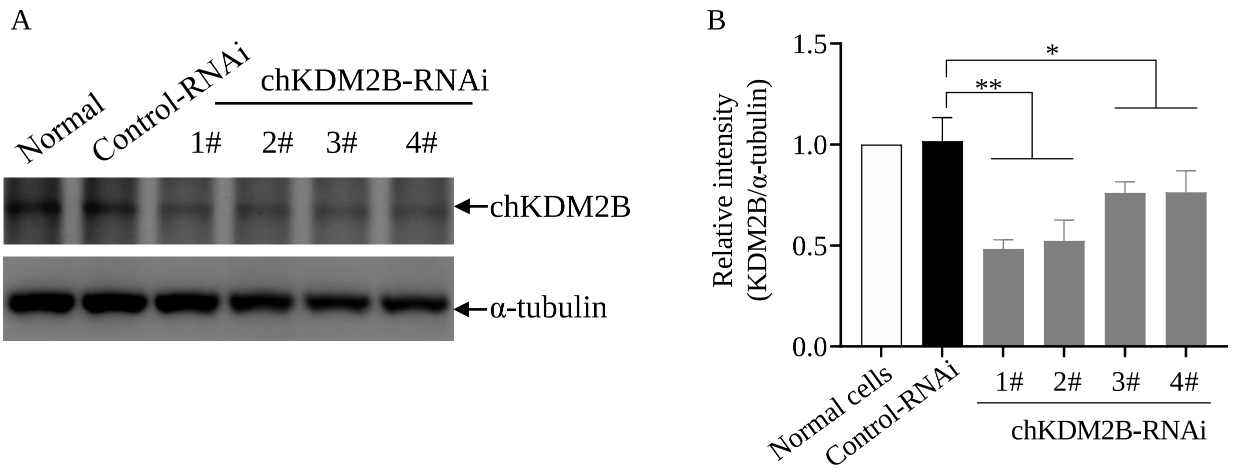

S3 Fig Effects of KDM2B RNAi on the expression of endogenous KDM2B in MSB1 cells. MSB1 cells were transfected with 50 nM siRNA constructs (1#-4#), RNAi controls or medium only. (A) Forty-eight hours after transfection, KDM2B protein expression was examined by Western blotting using anti-KDM2B antibodies, and (B) the band densities of KDM2B in panel A were quantitated by densitometry. Endogenous α-tubulin expression was examined as an internal control for Western blotting. The relative levels of KDM2B were calculated as follows: band density of KDM2B/that of α-tubulin. Data are representative of three independent experiments and presented as means ± SD. **, $P< 0.01$; *, $P< 0.05$.

S4 Fig

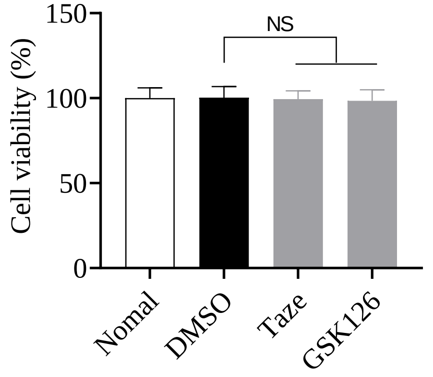

S4 Fig Effects of H3K27me3 inhibitors on the cell viability of MSB1 cells. MSB1 cells were treated with 5 μM Tazemetostat (Taze), 5 μM GSK126, or equal volume of dimethyl sulfoxide (DMSO) as a control. At 48 h after treatment, cell viability of MSB1 cells was detected by CCK8 assay. Data are representative of three independent experiments with three replicates and presented as means ± SD.
