# Supplementary material for: Marek’s disease virus-encoded microRNA-M6-5p facilitates viral latent infection by targeting histone demethylase KDM2B
Source: J Virol. 2025 Jan 22;99(2):e02007-24. doi: 10.1128/jvi.02007-24 (PMC11853111; doi:10.1128/jvi.02007-24)
Supplement: Table S1 — Sequences of oligonucleotides used in this study. [file jvi.02007-24-s0002.docx]

**S1 Table** . Sequences of oligos used in this study ^a^

| Name | Sequence (5’ -3’) | Application |
| --- | --- | --- |
| M6-galk-F | AGGAGATTTCCCGGTTTCGACTGCCGAAGC  ATGGAAACGTCCTGGGAAAACCTGTTGAC  AATTAATCATCGGCA | Amplification of galk  cassette gene |
| M6-galk-R | TAATTCCCAGAGAAAACTGTCATTTCGCA  GGGATCTCGAGTTAGTGTCACTCAGCAC  TGTCCTGCTCCTT |  |
| M6-galk-DE-F | AGGAGATTTCCCGGTTTCGACTGCCGAA  GCATGGAAACGTCCTGGGAAAAGTGACA  CTAACTCGAGATCCCTGCGAAATGACAGT  TTTCTCTGGGAATTA | Deletion  of galk  cassette gene |
| M6-galk-DE-R | TAATTCCCAGAGAAAACTGTCATTTCGC  AGGGATCTCGAGTTAGTGTCACTTTTCC  CAGGACGTTTCCATGCTTCGGCAGTCGAA  ACCGGGAAATCTCCT |  |
| M6-KanS-F | AGGAGATTTCCCGGTTTCGACTGCCGAA  GCATGGAAACGTCCTGGGAAAAAGGAT  GACGACGATAAGTAG | Amplification of I-SceI-Kan  cassette gene |
| M6-KanS-R | TAATTCCCAGAGAAAACTGTCATTTCGCA  GGGATCTCGAGTTAGTGTCACTTTTCCCA  GGACGTTTCCATGCTTCGGCAGTCGAAA  CCGGGAAATCTCCTCAACCAATTAACCA  ATTCTGATTAG |  |
| M6-F | ATCCCTGCATGATCTTCTTTA | Amplification of miR-M6 |
| M6-R | GCAGTTCTGAGGACACATTT |  |
| meq-F | ATGTCTCAGGAGCCAGAG | Amplification of meq |
| meq-R | TCAGGGTCTCCCGTCACC |  |
| meq-Q-F1 | CCCAACAGCCCCTCCAAACAC | Quantification of MDV |
| meq-Q-R1 | CTTCATGGAGTTTGTCTACA |  |
| ICP4-F | CGTGTTTTCCGGCATGTG | RT-qPCR |
| ICP4-R | TCCCATACCAATCCTCATCCA |  |
| UL36-F | GACAAGCTACTACAAATTGCA |  |
| UL36-R | GACGTCGATTTATCTCTTAACA |  |
| pp38-F | GAGCTAACCGGAGAGGGAGA |  |
| pp38-R | CGCATACCGACTTTCGTCAA |  |
| meq-F2 | TTGTCATGAGCCAGTTTGCCCTAT |  |
| meq-R2 | AGGGAGGTGGAGGAGTGCAAAT |  |
| vTR-F | CCTAATCGGAGGTATTGATGGTACTG |  |
| vTR-R | CCCTAGCCCGCTGAAAGTC |  |
| KDM2B-F | CTCGCAGCTAACCGGACCAC |  |
| KDM2B-R | GTGACACTCGCCGCACTCTG |  |
| GAPDH-F | TGCCATCACAGCCACACAGAAG |  |
| GAPDH-R | ACTTTCCCCACAGCCTTAGCAG |  |
| KDM2B-3’UTR-F | ATTCTAGGCGATCGCTCGAGCGAGGCGGG  GAAGGGACT | Construction of psiCHECK-2-KDM2B-3’UTR |
| KDM2B-3’UTR-R | TTATTGCGGCCAGCGGCCGCTCGCCTTATG  AATTTGCA |  |
| EP300-3’UTR-F | GTAATTCTAGGCGATCGCTCGAGAAAAAAA  GAAAAAGA | Construction of psiCHECK-2-EP300-3’UTR |
| EP300-3’UTR-R | TTATTGCGGCCAGCGGCCGCATTCTTTGTAC  TCTACTG |  |
| RBBP4-3’UTR-F | ATTCTAGGCGATCGCTCGAGCACAGCTGTAG  TGGAAGA | Construction of psiCHECK-2-RBBP4-3’UTR |
| RBBP4-3’UTR-R | TTATTGCGGCCAGCGGCCGCGATCCTGTAGC  CAGGATA |  |
| DHX38-3’UTR-F | ATTCTAGGCGATCGCTCGAGGCCTAAGGGCC  GAGAGGA | Construction of psiCHECK-2-DHX38-3’UTR |
| DHX38-3’UTR-R | TTATTGCGGCCAGCGGCCGCACCATAATGTC  CTTGAGC |  |
| METTL6-3’UTR-F | ATTCTAGGCGATCGCTCGAGGAAGAAAAGA  AAATCACC | Construction of psiCHECK-2-METTL6-3’UTR |
| METTL6-3’UTR-R | TTATTGCGGCCAGCGGCCGCCCAAAACCCG  ATCTTTTG |  |
| KDM2B-3’UTR-mut-F | CGAGAACTTGTTGAAACTACCCACGTTA  AGAATCT | Construction of psiCHECK-2-KDM2B-3’UTR-mut |
| KDM2B-3’UTR-mut-R | GTAGTTTCAACAAGTTCTCGACAAGAG  TTGTAAAA |  |
| vTR(0.3 kb)-F | GCTAAATCCAGGGCGGGAAA | ChIP-qPCR |
| vTR(-0.3 kb)-R | TTTGGAACTCCGCGGTCATT |  |
| vTR(-0.6 kb)-F | GGGCGATAAGACACTTTCCC |  |
| vTR(-0.6 kb)-R | TATGTGCCGGTTCCAGTGTG |  |
| vTR(-1.2 kb)-F | ACAAAACACTTACCCTCTCAACT |  |
| vTR(-1.2 kb)-R | CGTTCCTGATTTCCTTCCGC |  |
| pp38(-0.3 kb)-F | GCGGTATAGGATAAGAGATCAC |  |
| pp38(-0.3 kb)-R | GCGAAGAAGTTCCAAACGAT |  |
| pp38(-0.6 kb)-F | AGAGAAGGAACCTCGCAACC |  |
| pp38(-0.6 kb)-R | ATCACGTGACATGTACCGCC |  |
| pp38(-1.2 kb)-F | GTCGATGTTGACGGTGGTCT |  |
| pp38(-1.2 kb)-R | GATCTCAATTAATAGAACGGCGAT |  |
| miR-M6-3p | GAGAUCCCUGCGAAAUGACAGU | miRNA mimics |
| miR-M6-5p | UCUGUUGUUCCGUAGUGUUCUC |  |
| miR-M7-3p | UCGAGAUCUCUACGAGAUUACAG |  |
| miR-M7-5p | UGUUAUCUCGGGGAGAUCCCGAU |  |
| miR-M8-3p | GUGACCUCUACGGAACAAUAGU |  |
| miR-M8-5p | UAUUGUUCUGUGGUUGGUUUCG |  |
| miR-M10-3p | UCGAAAUCUCUACGAGAUAACA |  |
| miR-M10-5p | GCGUUGUCUCGUAGAGGUCCAG |  |
| miR-M13 | GCAUGGAAACGUCCUGGGAAA |  |
| miRNA/RNAi controls | UUCUCCGAACGUGUCACGUTT |  |
| miR-M6-5p inhibitor | GAGAACACUACGGAACAACAGA | miRNA inhibitors |
| miRNA inhibitor controls | CAGUACUUUUGUGUAGUACAA |  |
| KDM2B-RNAi 1# | GCUCUCAGAGUUCCACUUATT | RNAi |
| KDM2B-RNAi 2# | GCCAAAGAAUUGAACUGAATT |  |
| KDM2B-RNAi 3# | GCGAGUGUCACUUCUGCAATT |  |
| KDM2B-RNAi 4# | GCUGCAACGGGUGUACUUUTT |  |

^a^ F: forward primer, R: reverse primer
